# CCL2/CCR2 system in neuroepithelial radial glia progenitor cells: involvement in stimulatory, sexually dimorphic effects of maternal ethanol on embryonic development of hypothalamic peptide neurons

**DOI:** 10.1186/s12974-020-01875-5

**Published:** 2020-07-10

**Authors:** Guo-Qing Chang, Olga Karatayev, Devi Sai Sri Kavya Boorgu, Sarah F. Leibowitz

**Affiliations:** The Rockefeller University, 1230 York Avenue, New York, NY 10065 USA

**Keywords:** Maternal ethanol administration, Radial glia progenitor cells, Neuroimmune-neuropeptide interactions, CCL2/CCR2 chemokine system, Melanin-concentrating hormone (MCH) neurons, Neuroepithelium, Lateral hypothalamus, Sexual dimorphism

## Abstract

**Background:**

Clinical and animal studies show that alcohol consumption during pregnancy produces lasting behavioral disturbances in offspring, including increased alcohol drinking, which are linked to inflammation in the brain and disturbances in neurochemical systems that promote these behaviors. These include the neuropeptide, melanin-concentrating hormone (MCH), which is mostly expressed in the lateral hypothalamus (LH). Maternal ethanol administration at low-to-moderate doses, while stimulating MCH neurons without affecting apoptosis or gliogenesis, increases in LH the density of neurons expressing the inflammatory chemokine C-C motif ligand 2 (CCL2) and its receptor CCR2 and their colocalization with MCH. These neural effects associated with behavioral changes are reproduced by maternal CCL2 administration, reversed by a CCR2 antagonist, and consistently stronger in females than males. The present study investigates in the embryo the developmental origins of this CCL2/CCR2-mediated stimulatory effect of maternal ethanol exposure on MCH neurons.

**Methods:**

Pregnant rats from embryonic day 10 (E10) to E15 during peak neurogenesis were orally administered ethanol at a moderate dose (2 g/kg/day) or peripherally injected with CCL2 or CCR2 antagonist to test this neuroimmune system’s role in ethanol’s actions. Using real-time quantitative PCR, immunofluorescence histochemistry, in situ hybridization, and confocal microscopy, we examined in embryos at E19 the CCL2/CCR2 system and MCH neurons in relation to radial glia progenitor cells in the hypothalamic neuroepithelium where neurons are born and radial glia processes projecting laterally through the medial hypothalamus that provide scaffolds for neuronal migration into LH.

**Results:**

We demonstrate that maternal ethanol increases radial glia cell density and their processes while stimulating the CCL2/CCR2 system and these effects are mimicked by maternal administration of CCL2 and blocked by a CCR2 antagonist. While stimulating CCL2 colocalization with radial glia and neurons but not microglia, ethanol increases MCH neuronal number near radial glia cells and making contact along their processes projecting into LH. Further tests identify the CCL2/CCR2 system in NEP as a primary source of ethanol’s sexually dimorphic actions.

**Conclusions:**

These findings provide new evidence for how an inflammatory chemokine pathway functions within neuroprogenitor cells to mediate ethanol’s long-lasting, stimulatory effects on peptide neurons linked to adolescent drinking behavior.

## Background

There is strong clinical and preclinical evidence showing that maternal consumption or administration of ethanol during pregnancy has marked effects on brain development and behavioral function in offspring, contributing to an increased risk for alcohol use disorder [1–5]. While chronic ethanol at higher doses causes morphological and functional defects in neural development throughout the brain [6, 7], lower doses actually stimulate neurogenesis while having minimal impact on apotosis and astrocytes [8–11]. Maternal intraoral administration of ethanol in rat, at low-to-moderate doses from embryonic day 10 (E10) to E15 during peak hypothalamic neurogenesis, increases in the offspring the density of neurons in the lateral hypothalamus (LH) that express the orexigenic neuropeptide, melanin-concentrating hormone (MCH) [11, 12], which is known to have a role in reward and motivated behavior [13, 14]. This stimulatory effect on MCH is accompanied by behavioral changes, including increased alcohol consumption [11, 15], which are also induced in rats by central injection of MCH itself [16–18].

With ethanol exposure also stimulating inflammatory chemokines in different brain regions [19–22], these MCH neurons in the LH are of particular interest since they colocalize the inflammatory chemokine C-C motif ligand 2 (CCL2) and its primary receptor CCR2 [23]. Our recent studies demonstrate in adolescent offspring that maternal ethanol, while increasing neurogenesis, stimulates CCL2 and CCR2 and their expression in almost all MCH neurons, together with an increase in alcohol drinking [11, 15]. These neural and behavioral effects are reproduced by maternal administration of CCL2 and blocked by a CCR2 receptor antagonist, supporting the involvement of CCL2/CCR2 signaling in mediating ethanol’s actions. They are also sexually dimorphic during adolescence, consistently stronger in females than males. Of particular note is that these effects of ethanol in the LH of adolescent offspring are similarly detected in the embryo, where maternal ethanol exposure similarly increases the density of CCL2 and CCR2 cells and their colocalization in MCH neurons more consistently in females and these effects are blocked by maternal administration of a CCL2 antibody and CCR2 receptor antagonist, suggesting an important role for this neuroimmune system in the early development of MCH neurons in the LH [12].

The goal of this study was to investigate where and how ethanol acts in the embryo to affect the development of both the CCL2/CCR2 system and MCH neurons. We focused our analysis on the hypothalamic neuroepithelium (NEP) along the third ventricle where neurons are born [24]. In this area, we examined the radial glia progenitor cells, which play fundamental roles in patterning and differentiation of the developing CNS and are pluripotent, serving as primary progenitors to both neurons and glia including astroglia [25, 26]. They additionally provide long extended processes or scaffolds that guide migrating neurons from their site of differentiation to their terminal locations, thereby determining their density, distribution, and patterning [24, 27–29]. While high doses of ethanol are shown to reduce the density and disturb the morphology of radial glia and disrupt their function in promoting the differentiation and migration of neurons [30, 31], we used here the radial glia marker, brain lipid-binding protein (BLBP), to examine how a lower dose of ethanol that stimulates neurogenesis affects these radial glia cells in the hypothalamic NEP and their processes that project laterally through the medial hypothalamus (mHYP) toward the LH. With studies showing the CCL2/CCR2 system to be expressed in neural progenitor cells of the spinal cord [32] and to stimulate neuronal differentiation and migration in other brain regions [33–35], we additionally examined after ethanol exposure the relation of this neuroimmune system to radial glia cells in the hypothalamic NEP, their processes in the mHYP, and the density and patterning of MCH neurons as they migrate from the NEP through the mHYP toward the LH.

The evidence described in this report suggests the involvement of the embryonic CCL2/CCR2 system in radial glia neuroprogenitor cells of the hypothalamic NEP in mediating ethanol’s stimulatory, sexually dimorphic effects in utero on the density of MCH neurons in the hypothalamus. The significance of these embryonic events lies in the evidence that they are long lasting, with studies in adolescent offspring prenatally exposed to ethanol [11, 15] showing MCH neurons to be markedly increased in the LH with almost all colocalizing CCL2 and CCR2, a neural change closely linked to disturbances in alcohol-related behaviors.

## Methods

All procedures were conducted in a fully accredited AAALAC facility (22 °C, 12:12-h light-dark cycle with lights off at 7 am), in accordance with protocols approved by The Rockefeller University Animal Care and Use Committee and consistent with the NIH Guide to the Care and Use of Laboratory Animals.

### Animals

Time-pregnant, Sprague-Dawley rats (220–240 g) (Charles River Breeding Laboratories, Hartford, CT) arrived at the facility on embryonic day 5 (E5) and were acclimated to the laboratory conditions until E10, at which time experiments began as described in detail below. In all experiments, rodent chow (LabDiet Rodent Chow 5001, St. Louis, MO) and filtered water were available ad libitum. As described in the Experimental Design section below, all female and male embryos were sacrificed at E19, the age before birth used in our prior study of the embryo [12] when MCH neurons in the LH first exhibit an adult-like pattern [36], with 1 male and 1 female pup taken from each litter and the number of rats/sex/group (*n* = 6–7) equal to the number of litters/group.

### Maternal administration of ethanol

Pregnant rats (*n* = 6–7/experiment) were intraorally administered, from E10–E15 when MCH neurons develop in the hypothalamus [36], either a 2 g/kg/day ethanol solution (30% v/v) (“Ethanol”) or a control solution of maltose-dextrin made isocaloric to the ethanol solution (“Control”) [9], with an additional group of pregnant rats that were untreated controls (“Untreated”). The daily dose of ethanol was split in half with all rats gavaged twice daily, with the first gavage of 1 g/kg occurring 2 h after the start of the dark cycle and the second gavage of this dose occurring 7 h later. In blood collected from the tail vein at 2 h after the morning ethanol gavage on E11, blood ethanol concentration (BEC) was measured using Analox GM7 Alcohol Analyzer (Lunenburg, MA, USA) and was elevated to ~ 80 mg/dL, consistent with previous reports [9, 37]. Since this moderate dose and short period of ethanol exposure in pregnant rats are not expected to produce the symptoms of dependence and withdrawal observed with much higher doses (9–15 g/kg/day) and longer periods of exposure in non-pregnant rats [38, 39] further tests after removal of ethanol were not performed.

### Maternal administration of CCL2

Building on our recent studies showing administration of CCL2 itself to mimic the stimulatory effects of ethanol on CCL2 and CCR2 in the LH and produce these effects more strongly in females [11, 12], we gave pregnant rats (*n* = 6–7/experiment) one daily injection of CCL2 (4 μg/kg/day, s.c.), from E10 to E15 at 4 h into the dark cycle, as compared to its vehicle Control (sterile water) or Untreated controls, and examined its effects in female embryos at E19. This dose of CCL2 was chosen based on our previously published study [11], which is the first to describe the effect of maternal administration of CCL2 on the offspring and to show prenatal administration of CCL2, at 4 as well as 8 μg/kg/day from E10 to E15, to have similar stimulatory effects on the expression and density of CCL2/CCR2 and MCH expression in neurons.

### Maternal administration of CCR2 antagonist

We additionally tested the effects of maternal administration of the CCR2 receptor antagonist INCB3344 (MedChem Express, Cat. # HY-50674), previously used in rat studies of inflammation and pain [40, 41]. In pregnant rats (*n* = 6–7/experiment), ethanol (2 g/kg/day) or its isocaloric control from E10 to E15 were intraorally administrated twice daily as described above. In addition, we injected 30 min after each ethanol administration either INCB3344 (1 mg/kg/day, s.c.) or its vehicle Control (sterile water) or gave no treatment (Untreated), and female embryos in these groups were examined at E19. This dose of INCB3344 was chosen based on our previously published studies [11, 12] showing it to be effective in blocking the stimulatory effects of ethanol on neurons in the LH of adolescent rats and E19 embryos while having no effect on the dam’s food intake, water intake, and body weight or on the litter size and pup’s body weight at birth.

### Quantitative real-time PCR

Quantitative real-time PCR (qRT-PCR) was used to measure the gene expression of brain lipid-binding protein (BLBP), a radial glia marker [42], along with CCL2, CCR2, and MCH in the NEP and mHYP of embryos. They were sacrificed at E19, their tails were collected for genotyping to determine sex, and their brains were immediately removed and placed on a microscope slide on top of an ice-filled petri dish, with the ventral surface facing up for slicing. The NEP and mHYP areas were combined for the qRT-PCR analysis, based on our preliminary tests showing them separately to yield similar responses to ethanol and them combined to provide a more consistent dissection and more tissue needed for qRT-PCR as described in our recent report [12]. This area, referred to in the text as “NEP+mHYP”, was dissected as follows using gem razor blades (American Safety Razor Co., Verona, VA). Two coronal cuts were made, with the anterior cut 0.5 mm caudal to the posterior edge of the middle optic chiasm (Coronal Plate 10, E20) [43] and the posterior cut 1.0 mm caudal to the anterior cut (Coronal Plate 10 to 13, E20). After putting this slice on a microscope glass with its posterior level plain facing up, the NEP+mHYP area was further microdissected, with a lateral cut made 0.6 mm bilateral to hypothalamic third ventricle, a ventral cut made at the dorsal edge of the medial eminence, and a dorsal cut made 0.1 mm ventral to the fornix.

Total RNA was then extracted from each microdissected sample, cDNA was synthesized, and qRT-PCR was performed, as previously described in our publications [9, 11, 44] and others [45, 46]. The primers for BLBP, CCL2, CCR2, and MCH, designed with ABI Primer Express Version 3.0 software from published sequences, and their concentrations are presented in Table 1. The mRNA levels of the target gene in each rat were normalized within subject relative to mRNA levels of the internal house-keeping gene, cyclophilin, in the same sample, and this ratio of target gene expression to house-keeping gene expression was calculated in each rat using the standard delta-delta Ct method. Then, target gene mRNA levels were averaged in each rat within control and ethanol groups, and the average of the target gene mRNA level in the ethanol group was compared to the level in control. An ANOVA was then run on this ratio calculated for each subject, with the effect of ethanol on target gene expression determined by comparing the average ratio in the experimental groups to the control groups, as well as the control groups with each other. The mRNA data presented in Tables 5 and 6 are an averaged ratio (target gene expression/house-keeping gene expression) in each group, and those presented in the bar graphs of Fig. 2 are presented as fold change for clarity [11, 12]. The variability we observed in the baseline measures of CCL2, CCR2, and BLBP mRNA levels in the NEP as shown in these tables may be real, as it was confirmed in two separate groups of control embryos, was similarly observed with measures of CCL2 and CCR2 in the LH of adolescent control groups [11, 12], and is consistent with our additional finding with immunofluorescence histochemistry (Table 7) showing the density of BLBP cells in the NEP to be considerably greater than that of the CCL2 cells in this area.

**Table 1:** Primers for measurements of mRNA levels using qRT-PCR

Gene	GenBank accession	Forward 5′-3′	Reverse 5′-3′	Concentration (nM)
Cyclophilin	NM_001004279	TGTGCTGAATATTGGTGCTTGTAA	TGTGCTGAATATTGGTGCTTGTAA	200
BLBP	NM_030832	TGATTCGGTTGGATGGAGACA	CGACATCCCCAAAGGTGAGA	200
CCL2	NM_031530	GTG CTG TCT CAG CCA GAT GCA GTT	AGT TCT CCA GCC GAC TCA TTG GG	200
CCR2	NM_021866	TAC CTG TTC AAC CTG GCC ATC T	AGA CCC ACT CAT TTG CAG CAT	200
MCH	M_029712	CAAACAGGATGGCGAAGATGA	AGGCTTTCCCCATCCTGAAT	50

*BLBP* brain lipid-binding protein, *CCL2* C-C motif ligand 2, *CCR2* receptor for CCL2, *MCH* melanin-concentrating hormone

### Single- and double-label immunofluorescence histochemistry

**Fig. 1:**
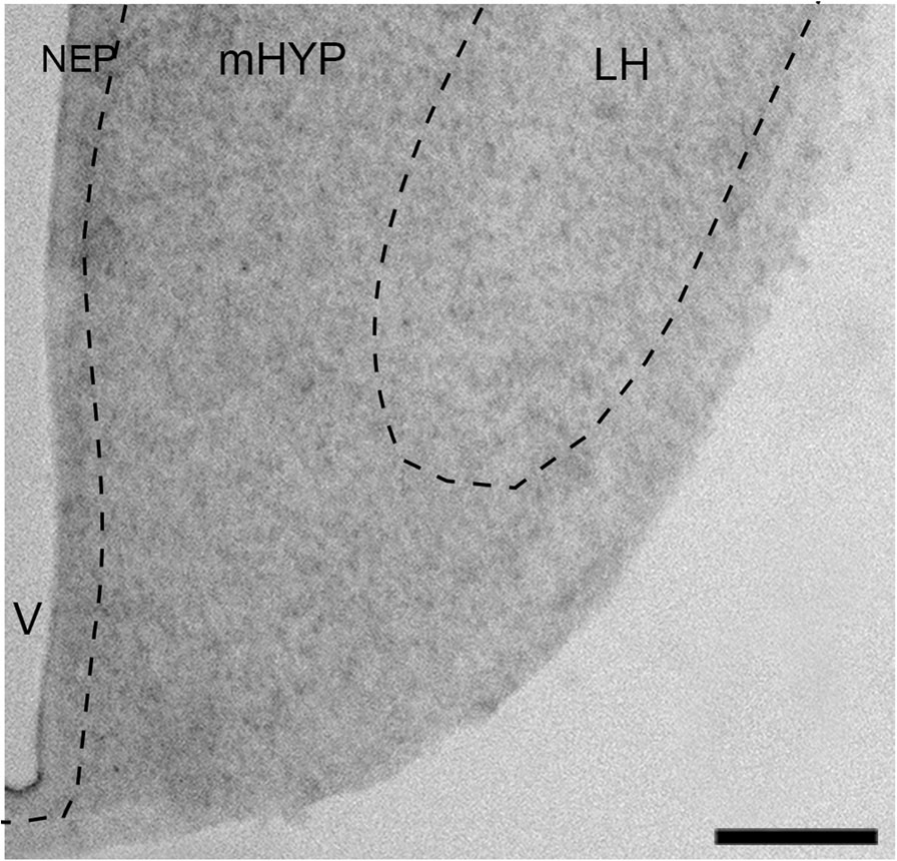
DIC image (× 2.5) illustrates in E19 embryo the three hypothalamic regions investigated in this manuscript. It shows from medial to lateral the hypothalamic neuroepithelium (NEP) along the third ventricle (V), the medal hypothalamus (mHYP) immediately lateral to the NEP, and the lateral hypothalamus (LH). Scale bar 200 μm

Immunofluorescence histochemistry (IF) was used, as previously described [11, 15, 47], to characterize the distribution pattern and quantify in the embryo at E19 the following: (1) BLBP-immunoreactive (BLBP^+^) radial glia progenitor cells; (2) CCL2-immunoreactive (CCL2^+^) cells in the NEP, with CCR2^+^ cells not detected here at E19 using available antibodies; (3) BLBP^+^ and CCL2^+^ fibers or processes of these NEP cells, which are seen in the mHYP as they project laterally toward the LH and are longer and more clearly defined for the BLBP^+^ than CCL2^+^ processes; and (4) MCH-immunoreactive (MCH^+^) neurons, which in the embryo can be detected although sparsely in the NEP, become denser in the mHYP, and are most dense in the LH. While we also tested GFAP, a marker for astrocytes, we observed few GFAP^+^ cells in the NEP and mHYP of ethanol-exposed and control embryos and no effect of maternal ethanol on these cells, as shown previously in adolescent offspring [11]. The precise areas examined, the NEP and mHYP as they relate to the LH, are illustrated in the DIC image of an embryonic brain section (Fig. 1).

To analyze at E19 the BLBP^+^, CCL2^+^, and MCH^+^ cells in the NEP and the BLBP^+^ and CCL2^+^ processes in the mHYP using single-labeling IF, the embryos were killed by perfusing their dams intracardially with 0.9% normal saline followed by 4% paraformaldehyde in phosphate buffer (PB), their tails were collected for genotyping to determine the sex, and their brains were removed and post-fixed for 4 h in the same fixative at 4 °C, cryo-protected in 25% sucrose at 4° C for 72–96 h, and then frozen and stored at − 80 °C. Brains were cut at 30 μm with a cryostat, and free-floating coronal sections were processed with the primary antibodies and their corresponding secondary antibodies listed in Table 2.

**Table 2:** Antibodies used for single-labeling immunofluorescence histochemistry

Primary antibody	Dilution	Vendor	Secondary antibody	Dilution	Vendor
Rabbit anti-CCL2	1:200	Biorbyt, CA	Bio-Horse anti-Rabbit IgG + TSA Fluorescein	1:100	Vector, CA
Rabbit anti-BLBP	1:200	Abcam, MA	Cy3-Donkey anti-Rabbit IgG	1:100	Jackson ImmunoResearch Laboratories Inc, PA
Rabbit anti-MCH	1:1000	Phoenix Pharmaceuticals, CA	Cy3-Donkey anti-Rabbit IgG	1:100	Jackson ImmunoResearch Laboratories. Inc, PA

*CCL2* C-C motif ligand 2, *BLBP* brain lipid-binding protein, marker for radial glia, *MCH* melanin-concentrating hormone

Sections were viewed, and fluorescence images were captured using a Zeiss LSM 880 confocal microscope with a × 20 objective. For quantitation, the images of the NEP and mHYP illustrated in Fig. 1 were outlined and analyzed. For each embryo, 8–10 images were collected at the anterior-posterior level of coronal plates 12 to 13 in E20 brains [43]. The density of single-label immunofluorescent cells in the NEP was then quantified, using Image-Pro Plus software (Version 4.5; Media Cybernetics) as previously described [11, 15, 47]. Only intact cells with an area of 50–100 μm^2^ were counted, and the population density of these cells in the NEP is reported as cells/μm^2^. The measure in the mHYP recorded as objects/μm^2^ reflects mostly the fibers of the BLBP^+^ and CCL2^+^ cells which we found to be dense in this area where few cells are evident, leading us to refer to these objects as “processes” in the text.

Double-labeling IF was used to determine whether CCL2 in the NEP and mHYP of the embryo colocalizes with the radial glial marker BLBP in its cells and processes, as well as with the neuronal marker NeuN and microglia marker Iba-1 in these cells, and also whether the MCH neurons are anatomically associated with the BLBP radial glia cells and processes. This double labeling was performed using a combination of primary antibodies and their corresponding secondary antibodies listed in Table 3, based on our procedures described previously [11, 15, 47].

**Table 3:** Antibodies used for double-labeling immunofluorescence histochemistry

Combination	Primary antibody	Dilution	Vendor	Secondary antibody	Dilution	Vendor
CCL2+BLBP	Rabbit anti-CCL2	1:200	Biorbyt, CA	Bio-Horse anti-Rabbit IgG + TSA Fluorescein	1:100	Vector, CA
	Mouse anti-BLBP	1:200	Abcam, MA	Cy3-Donkey anti-Mouse IgG	1:100	Jackson ImmunoResearch Laboratories. Inc, PA
CCL2+NeuN	Rabbit anti-CCL2	1:200	Biorbyt, CA	Bio-Horse anti-Rabbit IgG + TSA Fluorescein	1:100	Vector, CA
	Mouse anti-NeuN	1:100	EMD Millipore, MA	Cy3-Donkey anti-Mouse IgG	1:100	Jackson ImmunoResearch Laboratories. Inc, PA
CCL2+Iba-1	Rabbit anti-CCL2	1:200	Biorbyt, CA	Bio-Horse anti-Rabbit IgG + TSA Fluorescein	1:100	Vector, CA
	Goat anti-Iba-1	1:200	Abcam, MA	Cy3-Donkey anti-Goat IgG	1:100	Jackson ImmunoResearch Laboratories. Inc, PA
MCH+BLBP	Rabbit anti-MCH	1:1000	Phoenix Pharmaceuticals, CA	FITC-Donkey anti-Rabbit IgG	1:50	Jackson ImmunoResearch Laboratories. Inc, PA
	Mouse anti-BLBP	1:200	Abcam, MA	Cy3-Donkey anti-Mouse IgG	1:100	Jackson ImmunoResearch Laboratories. Inc, PA

*CCL2* C-C motif ligand 2, *BLBP* radial glia marker brain lipid-binding protein, *NeuN* neuronal marker, *Iba-1* microglia marker, *MCH* melanin-concentrating hormone

For this analysis of double-labeling, the images were captured by a Zeiss LSM 880 confocal microscope with × 20 objective, and the double-labeling was further confirmed by Z-stack sectioning with a × 40 oil-immersion lens, with the Z-stacks 30 μm thick and the step size of 0.7–0.8 μm for optimal stack collection and analysis. In all analyses, the cells and processes were counted in each section, and only the cells of a designated size (area of 50–100 μm) were counted. The double-labeled cells counted in × 20 images and double-labeled processes counted in × 40 images are reported as the percentage of total single-labeled cells or processes, respectively. Some variability in CCL2 staining, while not evident with single labeling, sometimes occurred when the CCL2 antibody was co-labeled with other antibodies, particularly for NeuN and Iba-1, but not for BLBP. To confirm that the BLBP^+^ and CCL2^+^ cells in the NEP are epithelial cells, we used DAPI to stain the nucleus. The double-labeling of cells with DAPI and BLBP or CCL2 in the NEP was confirmed by confocal Z-Stack sectioning, as demonstrated in Figs. 4 and 5 showing that almost all of the radial glia cells and CCL2 cells contain a nucleus and thus are epithelial in nature. To confirm that the BLBP^+^ and CCL2^+^ cells in the NEP are epithelial cells, we used DAPI to stain the nucleus. Confocal Z-Stack sectioning (30 μm thick and step size of 1.02 μm) of double-staining of DAPI with BLBP or CCL2 in the NEP shown in Figs. 4 and 5 that almost all of the radial glia cells and CCL2 cells contain a nucleus, confirming that they are epithelial in nature.

### Digoxigenin-labeled in situ hybridization histochemistry for MCH

We performed digoxigenin-labeled in situ hybridization histochemistry (DIG-ISH) to investigate MCH^+^ neurons in the NEP and mHYP of E19 embryos and to determine their anatomical relationship to the BLBP^+^ radial glia cells and processes in these areas. Briefly, as previously described [47], DIG-labeled RNA probes and 30 μm free-floating cryostat coronal sections were employed, and AP-conjugated sheep anti-digoxigenin Fab fragments (1:1000; Roche Diagnostics, Indianapolis, IN, USA) and NBT/BCIP (Roche Diagnostics) were used to visualize MCH-expressing neurons (see Fig. 8b). A negative control was performed with a sense probe, which revealed no MCH-expressing neurons in the area extending from the NEP to the LH, and a positive control was performed in the LH where MCH-expressing neurons are known to be most heavily expressed, as previously described [48, 49]. Sections were viewed on a Leitz microscope (× 10 objective). The images were captured with a Nikon DXM 1200 digital camera (Nikon, Tokyo, Japan) and analyzed using Image-Pro Plus software (Version 4.5, Media Cybernetics Inc., Silver Spring, MD, USA) on a gray-value scale from 1 to 255. In each animal, 8–10 sections at the same level were used to examine the NEP and mHYP, and the entire area was outlined and analyzed as described [11, 15, 47]. The population density was used to determine the cell density in these two areas, with a few MCH^+^ neurons detected in the NEP and a denser concentration seen in the mHYP.

To determine whether these MCH^+^ neurons in the NEP and mHYP are related to the radial glia cells and processes, we performed double-labeling by combining DIG-ISH for MCH^+^ neurons with IF for BLBP^+^ radial glia in these areas of the E19 embryo brain. After the MCH signal was first visualized in NBT/BCIP, the sections were briefly washed in 0.1 M Tris-HCl containing 0.1 M NaCl and 50 mM MgCl2 (pH 9.5) and PBS. They were then processed for BLBP IF as described above, and FITC-Donkey anti-Rabbit IgG (1:50, Jackson ImmunoResearch Laboratories, Inc. PA) was used to reveal BLBP labeling. To verify the specificity of the double-labeling, the following controls were performed (see Fig. 9a): (1) MCH sense probe negative control: sense probe was used for MCH DIG-ISH in the double-labeling, which revealed no MCH expression without affecting BLBP immunofluorescence; (2) no secondary fluorescence antibody control: the secondary antibody, FITC-Donkey anti-Rabbit IgG, was omitted in the double-labeling, which revealed no BLBP immunofluorescence without affecting MCH expression; and (3) positive control: the double-labeling was performed in the NEP, mHYP, and LH where both MCH and BLBP are heavily expressed. Double-labeling of MCH with BLBP cells in the NEP and of MCH cells with BLBP processes in the mHYP was quantified, as described above. In addition, the number of MCH cells positioned along and closely contacting BLBP processes in the mHYP as they projected laterally toward the LH were counted manually in each image, and the percentage of these neurons relative to the total number of MCH neurons in the mHYP was determined.

### Statistical analysis

All data were collected and analyzed in a blinded fashion using SPSS (Version 23), and they are presented as mean ± SEM. All graphs were prepared using the GraphPad Prism software (Version 6). Data in experiments using female and male embryos were analyzed using a two-way ANOVA, which tested between-subject main effects of maternal ethanol treatment and sex, and the interactions between these two factors. A significant interaction was interpreted using simple main effect analyses to test the differences between sexes as well as the differences within each sex. Data in experiments examining the effect of maternal treatment on bodyweight of dams and embryos, dam’s chow intake and litter size as well as those experiments where only female embryos were analyzed using a one-way ANOVA, which tested the effects of maternal administration of ethanol, CCL2 or CCR2 antagonist on the different measures of BLBP, CCL2 and MCH, followed by LSD post hoc tests. Paired *t* tests were performed to directly compare the effects of maternal treatment vs control groups within each sex and the effects of maternal ethanol to isocaloric control when only two groups in one sex were examined.

## Results

### Maternal ethanol administration increases BLBP mRNA in NEP+mHYP more strongly in female than male embryos

**Fig. 2:**
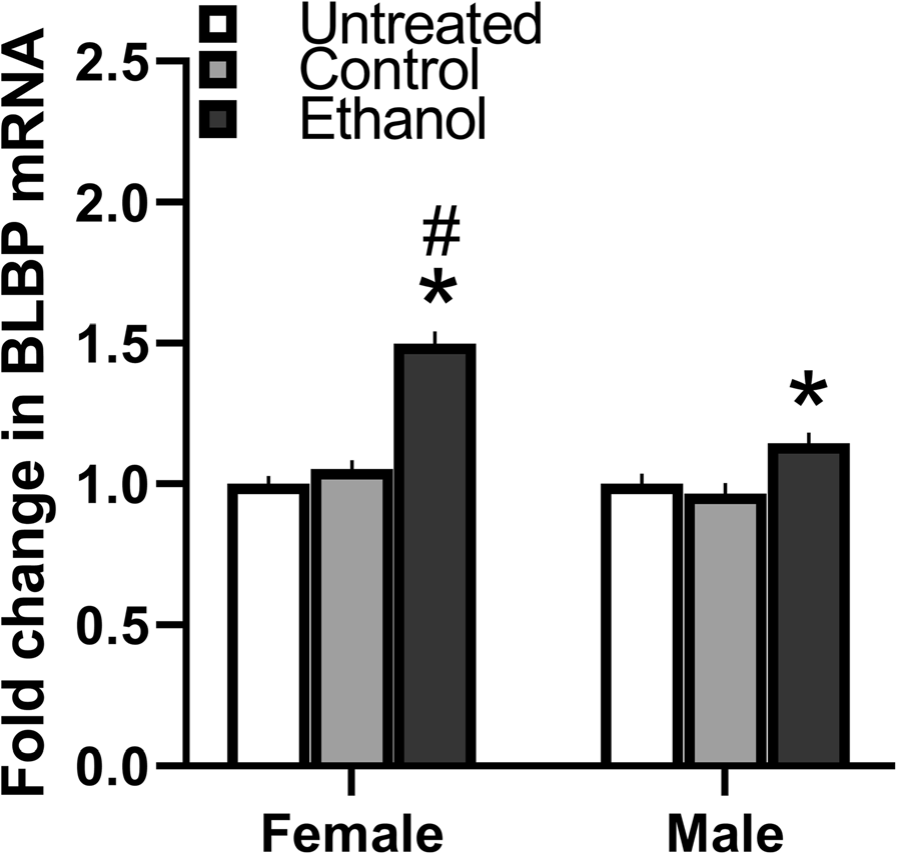
Effects of ethanol on expression of BLBP in NEP and mHYP. Maternal ethanol administration group (2 g/kg/day, E10–E15) was compared to Untreated and isocaloric Control groups of E19 embryos. Ethanol significantly increased in NEP+mHYP area the expression of BLBP measured using qRT-PCR, which is presented here as mRNA fold change compared to the Untreated control group. This effect on BLBP mRNA levels (with analysis of average ratio scores) was evident in both female embryos (Untreated = 0.223 ± 0.006, Control = 0.234 ± 0.007, Ethanol = 0.334 ± 0.014) and male embryos (Untreated = 0.221 ± 0.008, Control = 0.214 ± 0.008, Ethanol = 0.253 ± 0.009) but was significantly greater in females, with no differences between female and male control groups and between control groups within each sex. Data are mean ± SEM. (*n* = 7/group/sex, *p < 0.05 versus control group, ^#^*p* < 0.05 versus males). Two-way ANOVA was used to compare means between groups, simple main effect analyses to test differences between sexes as well as differences within each sex, and paired *t* tests to directly compare within each sex the effects of maternal treatment versus the control group.

Our first step was to determine whether maternal ethanol alters the expression of BLBP in the NEP+mHYP of embryos and whether this effect differs between the sexes. Using qRT-PCR, we tested the effects of maternal intraoral ethanol administration compared to Control and Untreated control groups of female and male embryos and found a significant main effect of ethanol treatment (*F*(2, 36) = 39.36, *p* < 0.001), in addition to an effect of sex (*F*(1, 36) = 20.73, *p <* 0.001) and a sex × treatment interaction (*F*(2, 36) = 9.96, *p* < 0.001) (Fig. 2). While there were no differences between respective control groups of females and males, the BLBP expression was higher in ethanol-exposed females compared to ethanol-exposed males (*p* < 0.001). Furthermore, ethanol significantly increased BLBP mRNA levels in both sexes compared to the Control (*p* < 0.001 for female and *p* = 0.004 for male) and Untreated (*p* < 0.001 for female and *p* = 0.018 for male) groups, with this effect significantly greater in females than males (*t*(12) = 3.49, *p* = 0.004). There was no significant main effect of maternal ethanol on the bodyweight of dams at E19 (*F*(2, 18) = 0.456, *p* = 0.641) and their E19 embryos (*F*(2, 18) =1.117, *p* = 0.349) and also no effect on dam’s daily chow intake (*F*(2, 18) = 1.156, *p* = 0.337) and litter size (*F*(2, 18) = 0.405, *p* = 0.405) (Table 4). Together, these results show that maternal ethanol administration at a moderate dose has a stimulatory effect on the expression of radial glia progenitor cells in the NEP+mHYP, with female embryos exhibiting greater sensitivity to ethanol.

**Table 4:** Body weights of embryos and dams and the chow intake and litter size of dams

Ethanol			
	Untreated	Control	Ethanol
Body weight—E19 (g)	6.670 ± 0.39	6.120 ± 0.16	6.310 ± 0.19
Body weight—dams (g)	295.5 ± 8.5	285.0 ± 12.3	282.1 ± 10.3
Chow intake (kcal/day)	71.90 ± 2.9	70.70 ± 3.2	65.40 ± 3.4
Litter size	11.70 ± 1.0	12.40 ± 0.7	12.70 ± 0.6
CCL2			
	Untreated	Control	CCL2
Body weight—E19 (g)	6.200 ± 0.28	6.000 ± 0.17	6.040 ± 0.18
Body weight—dams (g)	292.1 ± 7.4	277.3 ± 5.0	279.1 ± 6.5
Chow intake (kcal/day)	66.70 ± 4.1	68.40 ± 3.2	64.10 ± 4.4
Litter size	11.70 ± 1.2	12.40 ± 0.7	12.70 ± 0.6
Ethanol + INCB			
	Control	Ethanol	Ethanol + INCB3344
Body weight—E19 (g)	6.290 ± 0.23	6.070 ± 0.23	6.160 ± 0.13
Body weight—dams (g)	280.3 ± 8.7	273.9 ± 11.0	282.7 ± 9.1
Chow intake (kcal/day)	75.30 ± 4.7	72.20 ± 3.0	69.50 ± 2.7
Litter size	11.30 ± 1.0	11.60 ± 1.3	11.00 ± 1.2

Data are mean ± SEM. *n* = 7/group, *p < 0.05 versus control. Two-way ANOVA was used to compare means between groups and showed no significant effect of maternal administration of ethanol, CCL2 or CCR2 antagonist, INCB3344, on these measures

*kcal/day* kilocalories per day, *g* grams, *INCB3344* CCR2 antagonist

### Maternal ethanol administration stimulates radial glia cells in NEP and processes in mHYP more strongly in female than male embryos

**Fig. 3:**
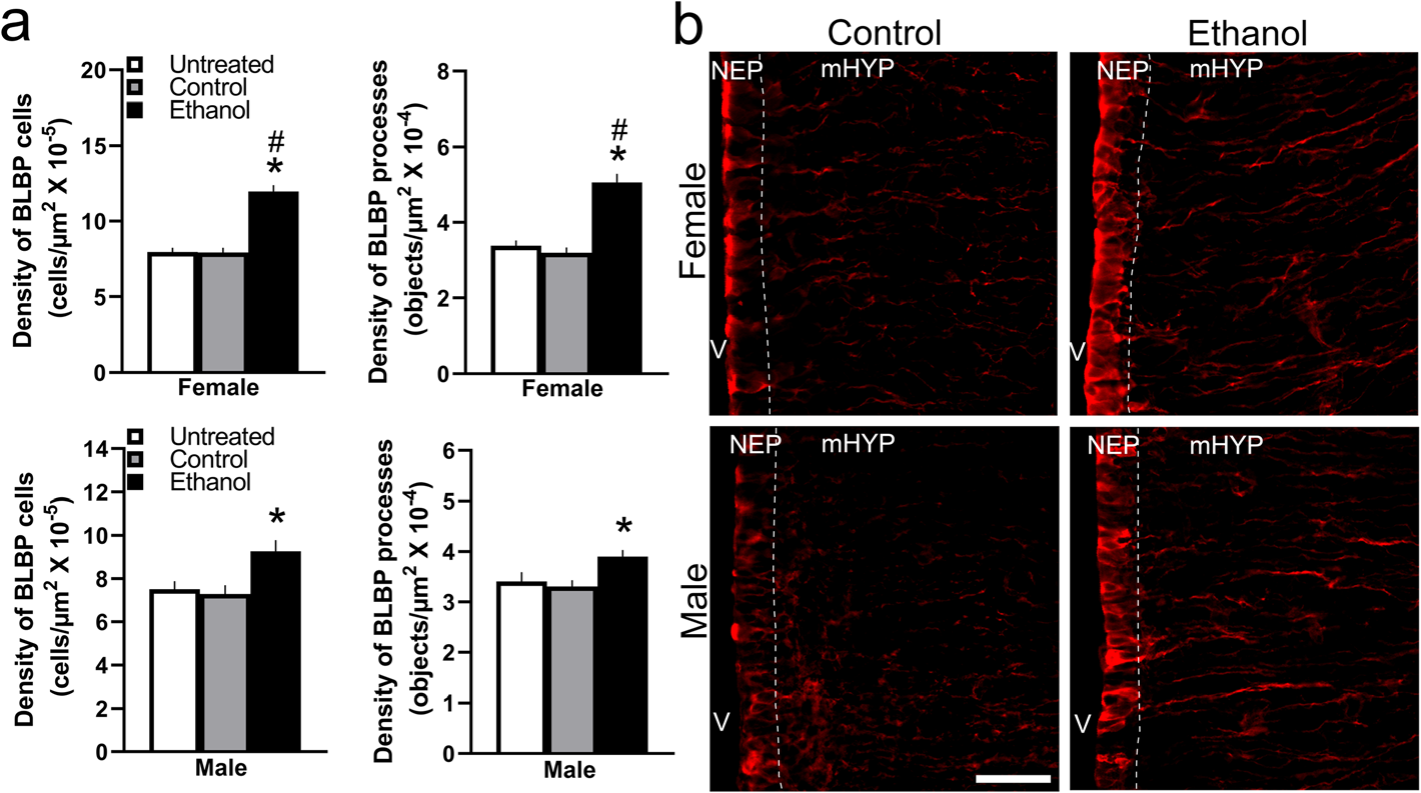
Effects of ethanol on the density of BLBP^+^ radial glia in NEP and mHYP. Maternal ethanol administration group (2 g/kg/day, E10–E15) was compared to Untreated and isocaloric Control groups of E19 embryos, and the density of cells and processes was examined using single-labeled immunofluorescence histochemistry. **a** Ethanol significantly increased the density of BLBP^+^ radial glia cells in NEP and of BLBP^+^ processes in mHYP of both female and male embryos, with females showing a significantly greater effect. **b** These effects in ethanol-exposed compared to control embryos are illustrated in representative immunostaining images (× 20) of single-labeled BLBP^+^ cells in NEP and BLBP^+^ processes in mHYP of female and male embryos. Data are mean ± SEM. (*n* = 7/group/sex, *p < 0.05 versus control group, ^#^*p* < 0.05 versus males). Two-way ANOVA was used to compare means between groups, simple main effect analyses to test the differences between sexes as well as differences within each sex, and paired *t* tests to directly compare the effects within each sex of maternal treatment vs control groups. BLBP, brain lipid-binding protein; mHYP, medial hypothalamus; NEP, neuroepithelium; V, third ventricle. Scale bars, 200 μm

We next wanted to determine whether maternal ethanol stimulates BLBP^+^ neuroprogenitor cells and whether this effect is sex-related. Using IF, we examined the effect of maternal administration of ethanol compared to Control and Untreated control groups on BLBP^+^ neuroprogenitor cells separately in the NEP and mHYP of female and male embryos. The BLBP^+^ radial glia cells were detected in the hypothalamic NEP and highly concentrated along the border of the third ventricle while very sparse in the mHYP. These cells have long, well-defined processes which project laterally through the mHYP in the direction of the LH. Maternal ethanol had strong stimulatory and sexually dimorphic effects on these cells and their processes. There was a significant main effect of ethanol treatment on the density of BLBP^+^ cells in the NEP (*F*(2, 36) = 38.54, *p* < 0.001) and BLBP^+^ processes in the mHYP (*F*(2, 36) = 34.69, *p* < 0.001), along with an effect of sex on BLBP^+^ cells (*F*(1, 36) = 16.10, *p* < 0.001) and processes (*F*(2, 36) = 34.69, *p* < 0.001) and a sex × treatment interaction for BLBP^+^ cells (*F*(2, 36) = 5.18, *p* = 0.011) and processes (*F*(2, 36) = 9.61, *p* < 0.001) (Fig. 3a), as illustrated in the photomicrographs (Fig. 3b). While there were no differences between respective control groups of females and males, ethanol-exposed females compared to ethanol-exposed males had a significantly higher density of the BLBP^+^ cells in the NEP (*p* < 0.001) and BLBP^+^ processes in the mHYP (*p* < 0.001). Also, maternal ethanol had a significant, stimulatory effect in both sexes on the density of BLBP^+^ cells in the NEP compared to the Control (*p* < 0.001 for female and *p* < 0.001 for male) and Untreated (*p* < 0.001 for female and *p* = 0.003 for male) groups and the density of BLBP^+^ processes in the mHYP compared to the Control (*p* < 0.001 for female and *p* = 0.013 for male) and Untreated (*p* < 0.001 for female and *p* = 0.036 for male) groups. However, the increase in density of BLBP^+^ cells was significantly greater in females than males compared to the Control (*t*(12) = 3.058, *p* = 0.010) and Untreated (*t*(12) = 5.0144, *p* = 0.021) groups, similar to the increase in density of BLBP^+^ processes compared to the Control (*t*(12) = 4.874, *p* < 0.001) and Untreated (*t*(12) = 3.353, *p* = 0.006) groups. Together, these results show that maternal ethanol administration at a moderate dose has a strong stimulatory effect on radial glia cells in the hypothalamic NEP and their processes in the mHYP, which are sexually dimorphic, consistently stronger in females than males.

### Maternal ethanol administration stimulates CCL2 and CCR2 mRNA in NEP and mHYP predominantly in female embryos

Building on our previously published finding that CCL2 and CCR2 expression is increased in the LH of embryos exposed to ethanol, we next determined whether maternal ethanol alters their expression in the NEP of embryos and whether this effect is similarly sex-related. Using qRT-PCR, we examined the effect of maternal ethanol administration compared to Control and Untreated control groups on gene expression of this chemokine and its receptor and found a significant main effect of ethanol treatment on CCL2 (*F*(2, 36) = 15.88, *p* < 0.001) and CCR2 (*F*(2, 36) = 22.23, *p* < 0.001), along with an effect of sex on CCL2 (*F*(1, 36) = 9.78, *p* = 0.003) and CCR2 (*F*(2, 36) = 17.99, *p* < 0.001) and a significant interaction between sex and ethanol treatment for CCL2 (*F*(2, 36) = 4.73, *p* = 0.015) and CCR2 (*F*(2, 36) = 4.22, *p* = 0.023) (Table 5). While again there were no differences between respective control groups of females and males, ethanol-exposed females exhibited a greater expression of CCL2 (*p* < 0.001) and CCR2 (*p* = 0.002) compared to males. Also, maternal ethanol administration compared to control groups significantly increased CCL2 mRNA levels in female embryos (*p* < 0.001 for Control and *p* < 0.001 for Untreated) but had no effect in male embryos (*p* = 0.067 for Control and *p* = 0.298 for Untreated). In addition, while ethanol significantly increased CCR2 mRNA levels in both sexes compared to their Control (*p* < 0.001 for female and *p* = 0.037 for male) and Untreated (*p* < 0.001 for female and *p* = 0.016 for male) groups, the ethanol-induced increase in CCR2 mRNA in females was significantly greater than in males (*t*(12) = 3.738, *p* = 0.003) (Table 5). Together, these results demonstrate that maternal ethanol increases the expression of CCL2 in female but not in male embryos and the expression of CCR2 in the females greater than the males.

**Table 5:** Effects of maternal ethanol administration on mRNA of CCL2 and CCR2 in NEP+mHYP area in E19 embryos

mRNA	Female			Male		
Expression	Untreated	Control	Ethanol	Untreated	Control	Ethanol
CCL2	1.2E− 3 ± 5.7E− 5	1.2E− 3 ± 5.1E− 5	1.9E− 3 ± 4.0E− 5*^#^	1.3E− 3 ± 6.1E− 5	1.3E− 3 ± 5.5E− 5	1.4E− 3 ± 5.7E− 5
CCR2	4.7E− 3 ± 2.3E− 4	4.5E− 3 ± 2.1E− 4	6.7E− 3 ± 3.1E− 4*^#^	4.6E− 3 ± 4.0E− 4	4.2E− 3 ± 2.3E− 4	5.0E− 3 ± 2.1E− 4*

Effect of maternal administration of ethanol (2 g/kg/day, E10–E15) on mRNA levels of CCL2 and CCR2 was measured using qRT-PCR. Data are expressed as an averaged ratio (target gene expression/house-keeping gene expression) in each group and presented as mean ± SEM (*n* = 7/group/sex, *p < 0.05 versus Control). Two-way ANOVA was used to compare means between groups, simple main effect analyses to test differences between sexes as well as differences within each sex, and paired *t* tests to directly compare within each sex the effects of maternal treatment vs control groups

*NEP* hypothalamic neuroepithelium, *mHYP* medial hypothalamus, *qRT-PCR* real-time quantitative PCR

^#^*p* < 0.05 vs males

### Maternal ethanol administration stimulates the density of CCL2 cells in NEP and processes in mHYP predominantly in female embryos

**Fig. 4:**
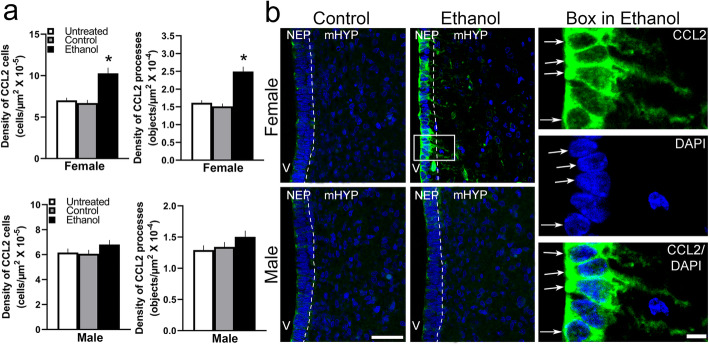
Stimulatory effects of ethanol on expression and density of CCL2^+^ cells in NEP and processes in mHYP. Maternal ethanol administration group (2 g/kg/day, E10–E15) was compared to Untreated and isocaloric Control groups of embryos at E19, and the density of cells and processes was examined using single-labeled immunofluorescence histochemistry. **a** Ethanol significantly increased the density of CCL2^+^ cells in NEP and CCL2^+^ processes in mHYP of female embryos, but it had no effect in male embryos. **b** These effects in ethanol-exposed female embryos are illustrated in representative immunostaining images (× 20) of double-labeled CCL2^+^ cells in NEP and their CCL2^+^ processes in mHYP with DAPI, with cells and processes in box illustrated to the right in × 40 images and both the CCL2^+^ cells and processes in male embryos (× 20 images) exhibiting no significant change. Data are mean ± SEM. (*n* = 7/group/sex, *p < 0.05 versus control group, ^#^*p* < 0.05 versus males). Two-way ANOVA was used to compare means between groups and simple main effect analyses to test differences between sexes as well as differences within each sex. NEP, neuroepithelium; mHYP, medial hypothalamus; V, third ventricle. Scale bars, 200 μm

The purpose of this experiment was to determine whether, in addition to stimulating the gene expression of CCL2, maternal ethanol exposure also alters the density of CCL2^+^ cells in E19 embryos. Using IF, we detected in E19 embryos a high concentration of CCL2^+^ cells in the NEP along the border of the third ventricle, with very few evident in the mHYP, and found these CCL2^+^ cells to be far denser in the NEP than the LH [12], with some having a distinct shape of short, lightly stained processes projecting laterally into the mHYP. Maternal ethanol administration had sexually dimorphic, stimulatory effects on both the CCL2^+^ cells and processes (Fig. 4a), as illustrated in the photomicrographs (Fig. 4b). There was a significant main effect of ethanol treatment on the density of CCL2^+^ cells in the NEP (*F*(2, 36) = 18.04, *p* < 0.001) and of CCL2^+^ processes in the mHYP (*F*(2, 36) = 23.30, *p* < 0.001), along with an effect of sex on CCL2^+^ cells (*F*(1, 36) = 28.50, *p* < 0.001) and processes (*F*(1, 36) = 39.46, *p* < 0.001) as well as a significant sex × treatment interaction on CCL2^+^ cells (*F*(2, 36) = 5.84, *p* < 0.001) and processes (*F*(2, 36) = 10.62, *p* < 0.001). While there were no differences between respective control groups of females and males, ethanol-exposed females had a significantly greater density of CCL2^+^ cells in the NEP (*p* < 0.001) and CCL2^+^ processes (*p* < 0.001) in the mHYP. Ethanol treatment significantly increased in females the density of CCL2^+^ cells in the NEP compared to the Control (*p* < 0.001) and Untreated (*p* < 0.001) groups, while having no effect on CCL2^+^ cells in the NEP of male embryos compared to the Control (*p* = 0.732) and Untreated (*p* = 0.080) groups. Similarly, ethanol also increased in females the density of CCL2^+^ processes in the mHYP compared to the Control (*p* < 0.001) and Untreated (*p* < 0.001) groups, while having no effect on the processes in the mHYP of male embryos compared to the Control (*p* = 0.251) and Untreated (*p* = 0.108) groups. Together, these results demonstrate a stimulatory effect of maternal ethanol on CCL2^+^ cells and processes in females but not males, similar to that shown for CCL2 mRNA and consistent with the stimulatory effect of ethanol on CCR2 mRNA that is stronger in females than males.

### Maternal administration of CCL2 stimulates BLBP^+^ and CCL2^+^ cells in the NEP and processes in the mHYP

**Fig. 5:**
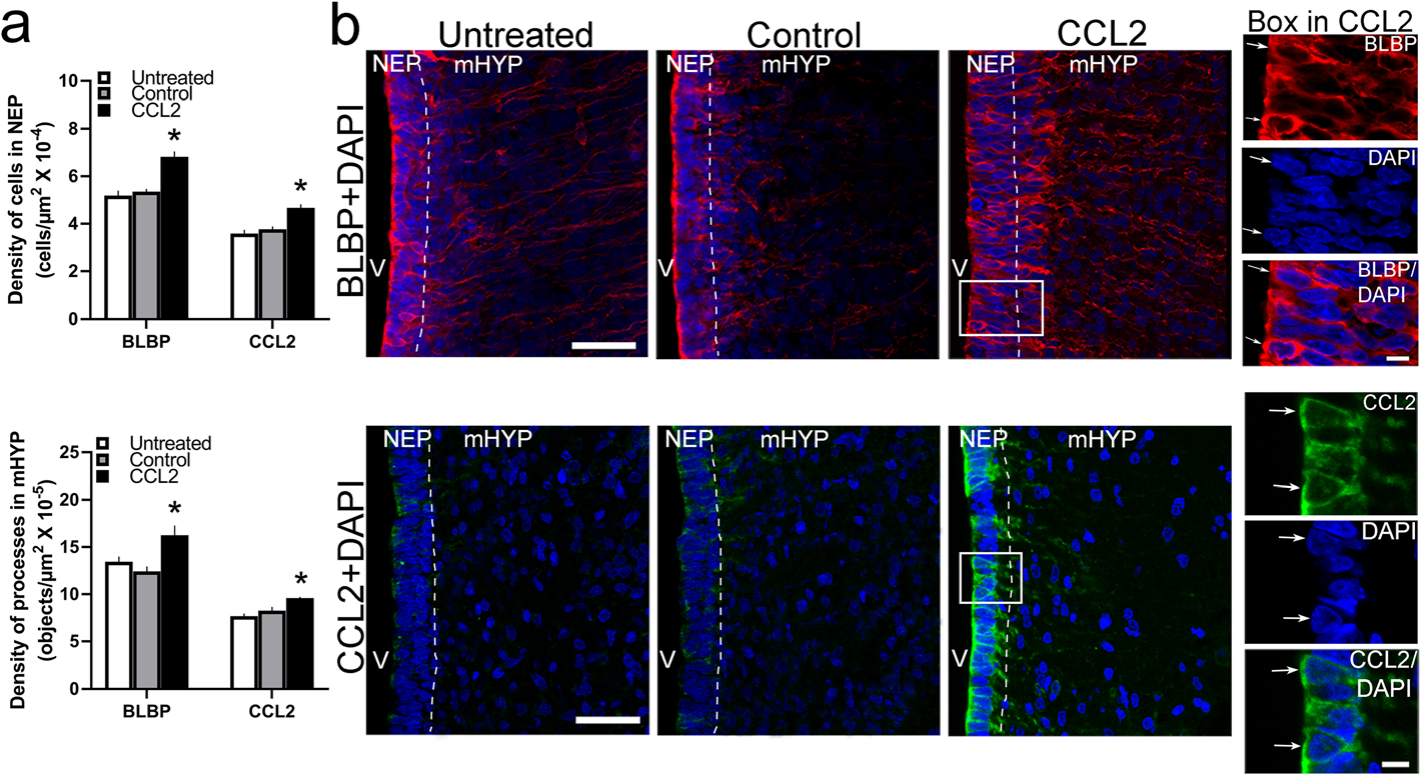
Stimulatory effects of CCL2 injection on BLBP^+^ and CCL2^+^ cells and processes in NEP and mHYP. Maternal administration of CCL2 (4 μg/kg/day, E10–E15) group was compared to Untreated and vehicle Control groups of female embryos at E19, and the density of cells and processes was analyzed using single-labeled immunofluorescence histochemistry. **a** CCL2 administration compared to control groups significantly increased the density of BLBP^+^ and CCL2^+^ cells in NEP and processes in mHYP. **b** These effects of ethanol compared to isocaloric Control group are illustrated in representative immunostaining images of double-labeled BLBP^+^ cells and processes (red) and CCL2^+^ cells and processes (green) with the nuclear stain DAPI (blue). Data are mean ± SEM. (*n* = 7/group, *p < 0.05 versus control group). One-way ANOVA was used to compare group means followed by SDS post hoc test. mHYP, medial hypothalamus; NEP, neuroepithelium; V, third ventricle. Scale bar, 100 μm

The purpose of this experiment was to determine whether CCL2 administration, similar to ethanol as shown above, also stimulates radial glia in the NEP and mHYP along with endogenous CCL2/CCR2 signaling. Using IF, we tested female E19 embryos from dams that were either untreated (Untreated) or given daily injections (s.c., E10–E15) of CCL2 at 4 μg/kg/day (CCL2) compared to sterile water vehicle (Control). There was a significant main effect of maternal CCL2 injection on the density of BLBP^+^ (*F*(2, 20) = 23.41, *p* < 0.001) and CCL2^+^ (*F*(2, 20) = 15.59, *p* < 0.001) cells in the NEP and of BLBP^+^ (*F*(2, 20) = 7.525, *p* = 0.004) and CCL2^+^ (*F*(2, 20) = 11.857, *p* < 0.001) processes in the mHYP (Fig. 5a), as illustrated in the photomicrographs (Fig. 5b). Maternal CCL2 significantly increased in the NEP the density of BLBP^+^ cells compared to Control (*p* < 0.001) and Untreated (*p* < 0.001) groups and of CCL2^+^ cells compared to Control (*p* < 0.001) and Untreated (*p* < 0.001) groups. It also increased the density of BLBP^+^ processes compared to Control (*p* < 0.001) and Untreated (*p* = 0.013) groups and of CCL2^+^ processes compared to Control (*p* = 0.004) and Untreated (*p* < 0.001) groups. Examination of the effect of maternal CCL2 on body weights revealed no significant main effect in the dams at E19 (*F*(2, 18) = 1.921, *p* = 0.175) or the E19 embryos (*F*(2, 18) = 0.251 *p* = 0.781) and also no effect on dam’s chow intake (*F*(2, 18) = 0.309, *p* = 0.738) and litter size (*F*(2, 18) = 0.405, *p* = 0.673) (Table 4). These results confirm that maternal administration of CCL2 mimics the effects of maternal ethanol, stimulating the cells and processes of both radial glia and CCL2 in the hypothalamic NEP and mHYP.

### Maternal administration of CCR2 antagonist blocks ethanol’s stimulatory effects on BLBP and CCL2 mRNA

With CCR2 shown to be involved in the stimulatory effect of maternal ethanol administration on CCL2 neurons in the LH [11, 12], we next tested the effect of a blockade of this receptor with maternal administration of the CCR2 receptor antagonist INCB3344 during the period of ethanol exposure on the radial glia and CCL2/CCR2 system in the NEP and mHYP. Using qRT-PCR, we examined female embryos at E19 in the following four groups: Isocaloric Control + Vehicle (Control); Ethanol + Vehicle (Ethanol); Ethanol + INCB3344; and Control + INCB3344. There was a significant main effect of treatment on mRNA levels of BLBP (*F*(3, 27) = 9.051, *p* < 0.001) and CCL2 (*F*(3, 27) = 14.795, *p* < 0.001) as well as CCR2 (*F*(3, 27) = 6.690, *p* = 0.002) in the NEP+mHYP area (Table 6). Maternal ethanol compared to Control significantly increased the expression of BLBP (+ 43%, *p* < 0.001) and CCL2 (+ 57%, *p* < 0.001) as well as of CCR2 (+ 49%, *p* = 0.002), and these effects were blocked by maternal administration of the CCR2 antagonist, causing a decrease in mRNA expression of BLBP (*p* < 0.001), CCL2 (*p* < 0.001), and CCR2 (*p* = 0.006) to the same levels as those in the Control group (*p =* 0.336, *p =* 0.641, and *p =* 0.483, respectively). The antagonist alone was found to have no effect of its own on these measures, with no differences evident between the Control + INCB3344 and the Control groups in their expression of BLBP (*p* = 0.956), CCL2 (*p* = 0.912), and CCR2 (*p =* 0.513). Consistent with our previous findings [15], maternal administration of CCR2 antagonist had no significant main effect on the body weight of dams at E19 (*F*(3, 24) = 0.315, *p* = 0.906) or their E19 embryos (*F*(3, 24) = 0.184, *p* = 0.906) and also no effect on the dam’s chow intake (*F*(3, 24) = 0.372, *p* = 0.774) and litter size (*F*(3, 24) = 0.405, *p* = 0.750) (Table 4). These findings support the involvement of the endogenous CCR2 receptor in mediating ethanol’s stimulatory effect on the radial glia and CCL2/CCR2 system in the NEP and mHYP.

**Table 6:** Effects of ethanol and CCR2 antagonist, INCB3344, on BLBP, CCL2, and CCR2 mRNA in NEP+mHYP area

mRNA expression	Control	Ethanol	Ethanol + INCB3344	Control + INCB3344
BLBP	2.3E− 1 ± 9.4E− 3	3.3E− 1 ± 2.2E− 2*	2.5E− 1 ± 1.9E− 2	2.3E− 1 ± 6.4E− 3
CCL2	1.3E− 3 ± 4.9E− 5	2.0E− 3 ± 1.5E− 4*	1.4E− 3 ± 6.9E− 5	1.2E− 3 ± 5.2E− 5
CCR2	4.5E− 1 ± 3.4E− 3	6.7E− 3 ± 5.4E− 4*	4.8E− 3 ± 4.1E− 4	4.1E− 3 ± 4.8E− 4

Maternal ethanol administration group (2 g/kg/day, E10–E15) with and without INCB3344 treatment (1 mg/kg/day, i.p.) was compared to Untreated and isocaloric Control groups of embryos at E19. Data are expressed as an averaged ratio (target gene expression/house-keeping gene expression) in each group and presented as mean ± SEM (*n* = 7/group, *p < 0.05 versus Control, Ethanol + INCB3344, and Control + INCB3344 groups). Difference between groups was analyzed using one-way ANOVA followed by SDS post hoc test

*BLBP* brain lipid-binding protein, *NEP* neuroepithelium

### Maternal administration of CCR2 antagonist blocks ethanol’s stimulatory effects on BLBP and CCL2 cells and processes

**Fig. 6:**
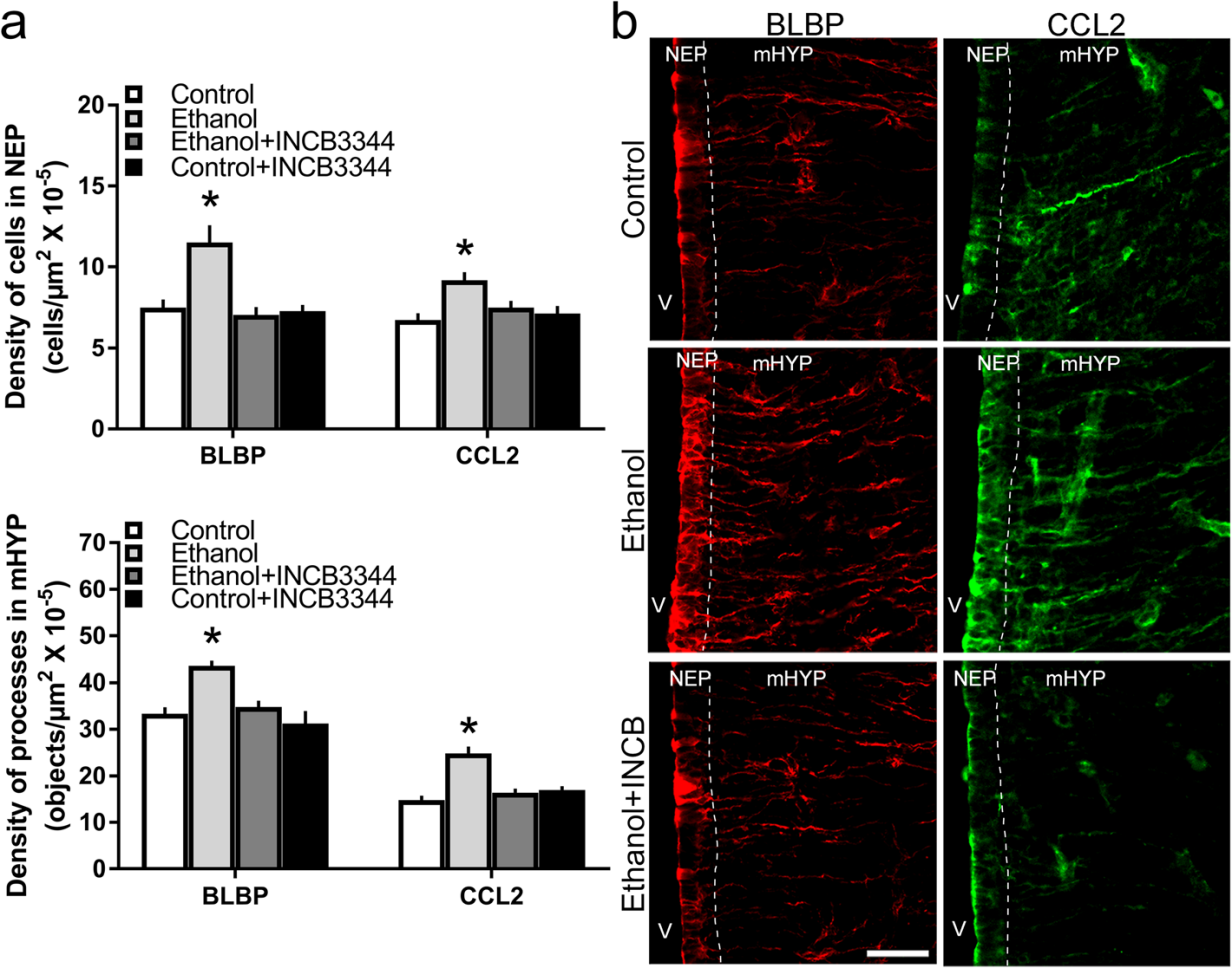
Impact of CCR2 receptor antagonist on ethanol’s effects on BLBP^+^ and CCL2^+^ cells and processes. Maternal injection of CCR2 receptor antagonist, INCB3344 (1 mg/kg/day, i.p.), was performed during the period of maternal ethanol administration (2 g/kg/day, E10–E15) and was compared to the Ethanol, Control, and Control + INCB3344 groups of E19 female embryos. **a** Analysis using single-labeled immunofluorescence histochemistry showed that Ethanol treatment compared to Control group significantly increased the density of BLBP^+^ and CCL2^+^ cells in NEP and processes in mHYP and the INCB3344 treatment during ethanol exposure blocked this effect, causing a significant decrease in the Ethanol + INCB3344 group compared to Ethanol group in cells and processes reduced to the same levels as those in the Control group. **b** These effects in the embryo are illustrated in representative images (× 20) of BLBP^+^ and CCL2^+^ single-labeled cells and processes in the Control, Ethanol, and Ethanol + INCB3344 embryos. Data are mean ± SEM (*n* = 7/group, *p < 0.05 versus the Control, Ethanol + INCB3344, and Control + INCB3344 groups). Differences between groups were analyzed using one-way ANOVA followed by SDS post hoc test. BLBP, brain lipid-binding protein; mHYP, medial hypothalamus; NEP, neuroepithelium; V, third ventricle. Scale bar, 100 μm

Building on our finding from the above experiment that CCR2 antagonist blocks ethanol-induced effect on BLBP and CCL2 mRNA, we wanted to determine here whether this effect extends to BLBP^+^ and CCL2^+^ cells and their processes. Using single-label IF, we examined female embryos at E19 from dams in the following four groups: Isocaloric Control + Vehicle (Control); Ethanol + Vehicle (Ethanol); Ethanol + INCB3344; and Control + INCB3344. We found a significant main effect of treatment on the density of BLBP^+^ (*F*(3, 27) = 10.228, *p* < 0.001) and CCL2^+^ (*F*(3, 27) = 5.572, *p* = 0.005) cells and of BLBP^+^ (*F*(3, 27) = 9.481, *p* < 0.001) and CCL2^+^ (*F*(3, 27) = 18.543, *p <* 0.001) processes (Fig. 6a), as illustrated in the photomicrographs (Fig. 6b). Ethanol compared to Control group caused a significant increase in the density of BLBP^+^ (+ 36%, *p* < 0.001) and CCL2^+^ (+ 53%, *p* < 0.001) cells in the NEP and of BLBP^+^ (+ 31%, *p* < 0.001) and CCL2^+^ (+ 67%, *p* < 0.001) processes in the mHYP. These effects were reversed by INCB3344 treatment, causing a significant decrease in the Ethanol + INCB3344 compared to Ethanol group in the density of BLBP^+^ cells (*p* = 0.004) and processes (*p* = 0.002) and CCL2^+^ cells (*p* = 0.006) and processes (*p <* 0.001) that were reduced to the same levels as those in the Control group for BLBP^+^ cells (*p =* 0.471) and processes (*p =* 0.639) and CCL2^+^ cells (*p =* 0.561) and processes (*p =* 0.310). There were no differences between the Control + INCB3344 group and the Control group in the measures of BLBP^+^ (*p* = 0.820) and CCL2^+^ (*p* = 0.538) cells and BLBP^+^ (*p* = 0.406) and CCL2^+^ (*p* = 0.164) processes. These results demonstrate the importance of CCR2 in mediating ethanol’s stimulatory effects on the cells and processes of radial glia in the NEP and mHYP.

### Maternal ethanol stimulates co-labeling of CCL2 in radial glia cells and neurons

Building on our published evidence that maternal ethanol stimulates CCL2 in the LH predominantly in neurons but not in microglia or astrocytes [12], we wanted in this experiment to determine the effect of maternal CCL2 on these neurons and radial glia in the NEP and mHYP. Using BLBP as the marker of radial glia, NeuN to label neurons, and Iba-1 to label microglia, we employed double-labeling IF to examine in female E19 embryos the co-labeling of CCL2 with BLBP, NeuN, and Iba-1. Maternal ethanol exposure stimulated all three cell types, radial glia, neurons, and microglia in the NEP and mHYP (Table 7). Confirming the above results, analyses of single-labeled cells and processes showed that maternal administration of ethanol compared to the Control group significantly increased the density of CCL2^+^ cells in the NEP (*p* = 0.014) and processes in the mHYP (*p* < 0.001) and of BLBP^+^ cells in the NEP (*p* = 0.002) and processes in the mHYP (*p* = 0.016). They further revealed a stimulatory effect of ethanol on the density of NeuN^+^ neurons, which were most concentrated along the periventricular region of the NEP (*p* = 0.010) while scattered throughout the mHYP (*p* = 0.026), and also of Iba-1^+^ microglia, which were detected along the lateral border of the NEP (*p* = 0.009) as well as scattered in the mHYP (*p* < 0.001) (Table 7).

**Table 7:** Effects of ethanol on density of cells or processes that label CCL2, BLBP, NeuN, or Iba-1 in NEP and mHYP

Labeled cells/processes	Location	Control (cells, objects/μm^2^)	Ethanol (cells, objects/μm^2^)
CCL2^+^	NEP	6.30E− 5 ± 2.67E− 6	7.50E− 5 ± 2.07E− 6*
	mHYP	9.90E− 5 ± 7.30E− 6	1.70E− 4 ± 1.46E− 5*
BLBP^+^	NEP	1.51E− 4 ± 4.62E− 6	1.83E− 4 ± 5.67E− 6*
	mHYP	1.59E− 4 ± 1.32E− 5	2.31E− 4 ± 2.13E− 5*
NeuN^+^	NEP	5.75E− 5 ± 2.77E− 6	7.92E− 5 ± 7.24E− 6*
	mHYP	4.28E− 5 ± 2.68E− 6	5.17E− 5 ± 2.64E− 6*
Iba-1^+^	NEP	3.79E− 5 ± 1.84E− 6	4.73E− 5 ± 1.79E− 6*
	mHYP	4.47E− 5 ± 2.14 E− 6	6.06E− 5 ± 4.27E− 6*

Maternal administration of ethanol (2 g/kg/day, E10–E15) was compared to an isocaloric maltose-dextrin control solution (Control) group in the NEP and mHYP areas of female E19 embryos, measured using single-labeling IF. Data are mean ± SEM, *n* = 6/group, *p < 0.05 versus Control. The difference between groups was analyzed using Student *t* test

*BLBP* brain lipid-binding protein, *CCL2* C-C motif ligand 2, *Iba-1* microglial marker, *mHYP* medial hypothalamus, *NEP* hypothalamic neuroepithelium, *NeuN* neuronal marker

**Fig. 7:**
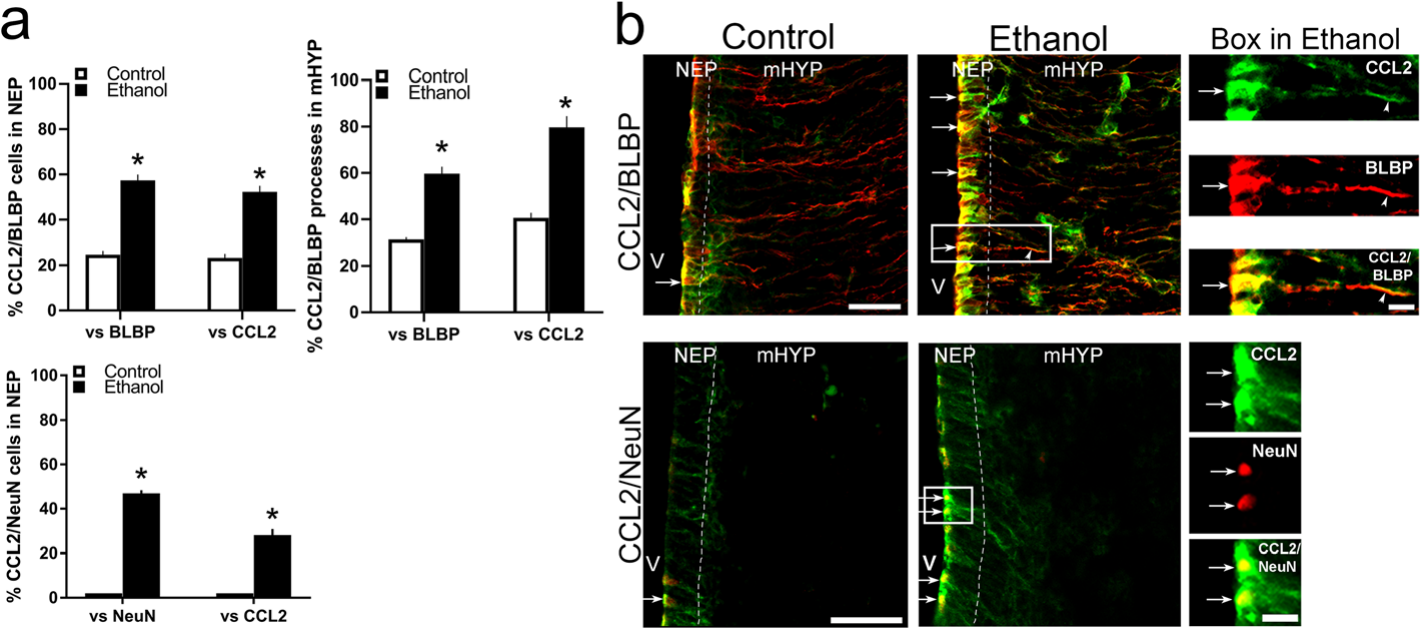
Stimulatory effects of ethanol on CCL2 in radial glia and neurons. Maternal ethanol administration (2 g/kg/day, E10–E15) was compared to isocaloric Control group with measurements of radial glia, neurons, and microglia in E19 female embryos. **a** Ethanol compared to Control group significantly increased the percentage of double-labeled CCL2^+^/BLBP^+^ radial glia cells relative to single-labeled BLBP^+^ or CCL2^+^ cells in NEP and double-labeled CCL2^+^/BLBP^+^ radial glia processes relative to single-labeled BLBP^+^ and CCL2^+^ processes in mHYP. It also increased the percentage of double-labeled CCL2^+^/NeuN^+^ neurons relative to single-labeled NeuN^+^ neurons and CCL2^+^ cells in NEP, with double-labeled CCL2^+^/NeuN^+^ neurons very sparse with no processes in the mHYP. **b** This effect of ethanol on double-labeled CCL2^+^/BLBP^+^ cells and processes and CCL2^+^/NeuN^+^ neurons is illustrated in × 20 images, with those in the box enlarged to the right (× 40) and showing (white arrows) the single-labeled CCL2^+^ (green) and BLBP^+^ or NeuN^+^ (red) cells and processes (white arrowheads) and the double-labeled CCL2^+^/BLBP^+^ cells and processes and CCL2^+^/NeuN^+^ cells (yellow). Data are mean ± SEM. (*n* = 6/group, *p < 0.05 versus control group). Differences between groups were analyzed using one-way ANOVA followed by SDS post hoc test. BLBP, brain lipid-binding protein; mHYP, medial hypothalamus; NEP, neuroepithelium; V, third ventricle. Scale bars, 100 μm

Analysis of the double-labeled cells and processes show CCL2 to colocalize with BLBP in radial glia and NeuN in neurons and maternal ethanol to stimulate their colocalization (Fig. 7a), as illustrated in the photomicrographs (Fig. 7b), but fail to reveal any colocalization of CCL2 with Iba-1 in microglia under any condition. Ethanol compared to Control significantly increased the percentage of double-labeled CCL2^+^/BLBP^+^ cells in the NEP relative to single-labeled BLBP^+^ (*t*(10) = − 32.05, *p* < 0.001) and CCL2^+^ (*t*(10) = − 10.21, *p* < 0.001) cells and of double-labeled CCL2^+^/BLBP^+^ processes in the mHYP relative to single-labeled BLBP^+^ (*t*(10) = − 10.374, *p* < 0.001) and CCL2^+^ (*t*(10) = − 8.359, *p* < 0.001) processes. These effects on double-labeled radial glia, evident in the × 20 photomicrographs of Ethanol compared to Control groups, are more clearly illustrated in the × 40 images to the right with arrowheads identifying the radial glia cells and processes (Fig. 7b). In addition, as described in the LH [11, 12]. Ethanol compared to Control significantly increased the percentage of double-labeled CCL2^+^/NeuN^+^ neurons in the NEP relative to single-labeled NeuN^+^ neurons (*t*(10) = − 32.046, *p* < 0.001) and CCL2^+^ cells (*t*(10) = − 10.220, *p* < 0.001), and these double-labeled neurons were concentrated along the periventricular border of the NEP while very sparse in the mHYP, as illustrated in the × 20 photomicrographs and identified by arrowheads in × 40 images to the right (Fig. 7b). This is in contrast to Iba-1^+^ microglia, which while stimulated by ethanol showed no double-labeling with CCL2. This analysis of CCL2^+^ cells in the hypothalamic NEP and mHYP demonstrates that, while few of the radial glia, neurons, and microglia contain CCL2 under control conditions, ethanol exposure stimulates all three cell types in the NEP and mHYP and increases the colocalization of CCL2 in ~ 60% of the radial glia and neurons while producing no colocalization in the microglia.

### Maternal ethanol increases MCH-expressing neurons in the NEP and mHYP of female more than male embryos

**Fig. 8:**
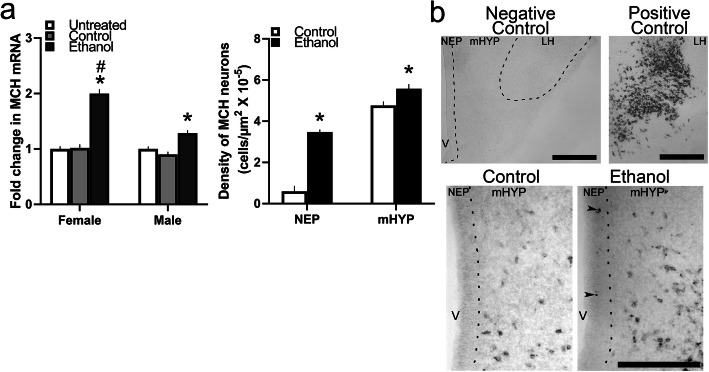
Stimulatory effects of ethanol on MCH expression and neurons in NEP and mHYP. Maternal ethanol administration group (2 g/kg/day, E10–E15) was compared to Untreated and isocaloric Control groups of embryos at E19. **a** Ethanol compared to control groups significantly increased in NEP+mHYP area mRNA expression of MCH measured using qRT-PCR which is presented here as mRNA fold change compared to Untreated control group. This effect on MCH mRNA levels (determined by analysis of average ratio scores) was evident in both female embryos (Untreated = 2.50 × 10^−3^, Control = 2.25 × 10^−3^, Ethanol = 4.99 × 10^−3^) and male embryos (Untreated = 2.62 × 10^−3^, Control = 2.36 × 10^−3^, Ethanol = 3.37 × 10^−3^) but was significantly greater in females. In a separate set of embryos, ethanol compared to the Control group (*n* = 6/group) significantly increased in females the density of MCH^+^ neurons in both the NEP and mHYP as measured using DIG-ISH. **b** This effect on MCH^+^ neurons is illustrated in representative × 10 images of MCH-expressing neurons revealed by DIG-ISH, with several neurons detected in the NEP of ethanol-treated embryos (two most distinct MCH^+^ neurons identified by black arrowheads) but none evident in Control embryos, and with the far denser population in the mHYP also significantly stimulated by ethanol (bottom panel). Top panel shows the negative and positive controls for MCH DIG-ISH. Data are mean ± SEM. (*n* = 7/group/sex, *p < 0.05 versus control group, ^#^*p* < 0.05 versus males). Two-way ANOVA was used to compare means between groups and simple main effect analyses to test differences between sexes as well as differences within each sex. mHYP, medial hypothalamus; MCH, melanin-concentrating hormone; NEP, neuroepithelium; V, third ventricle. Scale bar, 100 μm

This experiment investigated whether ethanol’s stimulatory effects on these CCL2 cells in the NEP are related to its effects on MCH neurons in the LH that colocalize CCL2 and CCR2 [11, 12, 15]. Embryos at E19 from two separate sets of dams were either untreated (Untreated) or received daily intraoral administration (from E10 to E15) of either 2 g/kg/day of ethanol (Ethanol) or an isocaloric maltose-dextrin control solution (Control) were examined. Measurements of MCH expression in the NEP+mHYP area of female and male embryos from the first set of dams revealed a significant increase in mRNA levels using qRT-PCR, albeit at much lower levels than found in the LH [12]. There was a significant main effect of ethanol treatment on MCH mRNA (*F*(2, 36) = 29.913, *p* < 0.001), in addition to an effect of sex (*F*(1, 36) = 9.933, *p* = 0.003) and a sex × ethanol treatment interaction (*F*(2, 36) = 5.018, *p* = 0.012) (Fig. 8a). While there were no differences between respective control groups of females and males, the ethanol-exposed females exhibited a significantly greater expression of MCH than the males (*p* < 0.001). Further, while ethanol significantly increased MCH mRNA levels in both sexes compared to their Control (*p* < 0.001 for female and *p* = 0.014 for male) and Untreated (*p* < 0.001 for female and *p* = 0.005 for male) groups, direct comparisons between females and males showed this effect of ethanol on MCH expression to be significantly greater in female than male embryos compared to the Control (*t*(12) = 2.802, *p* = 0.016) and Untreated (*t*(12) = 4.152, *p* < 0.001) groups (Fig. 8a). Analysis of these MCH^+^ neurons using DIG-ISH in the Ethanol compared to Control female embryos from the second set of dams revealed a significant increase in their density in the NEP (*t*(10) = − 9.76, *p* < 0.001) and mHYP (*t*(10) = − 2.69, *p* = 0.023) (Fig. 8a), as illustrated in the photomicrographs (Fig. 8b). A few MCH^+^ neurons were detected in the NEP, although only in ethanol-exposed embryos with a denser concentration of these neurons in the mHYP. Thus, similar to its effect in the LH [11], maternal ethanol significantly increased in the embryo mRNA expression of MCH, more strongly in females than males, and the density of MCH neurons in both the NEP and mHYP of females.

### Maternal ethanol increases the number and percentage of MCH neurons close to radial glia in NEP and mHYP of embryos

**Fig. 9:**
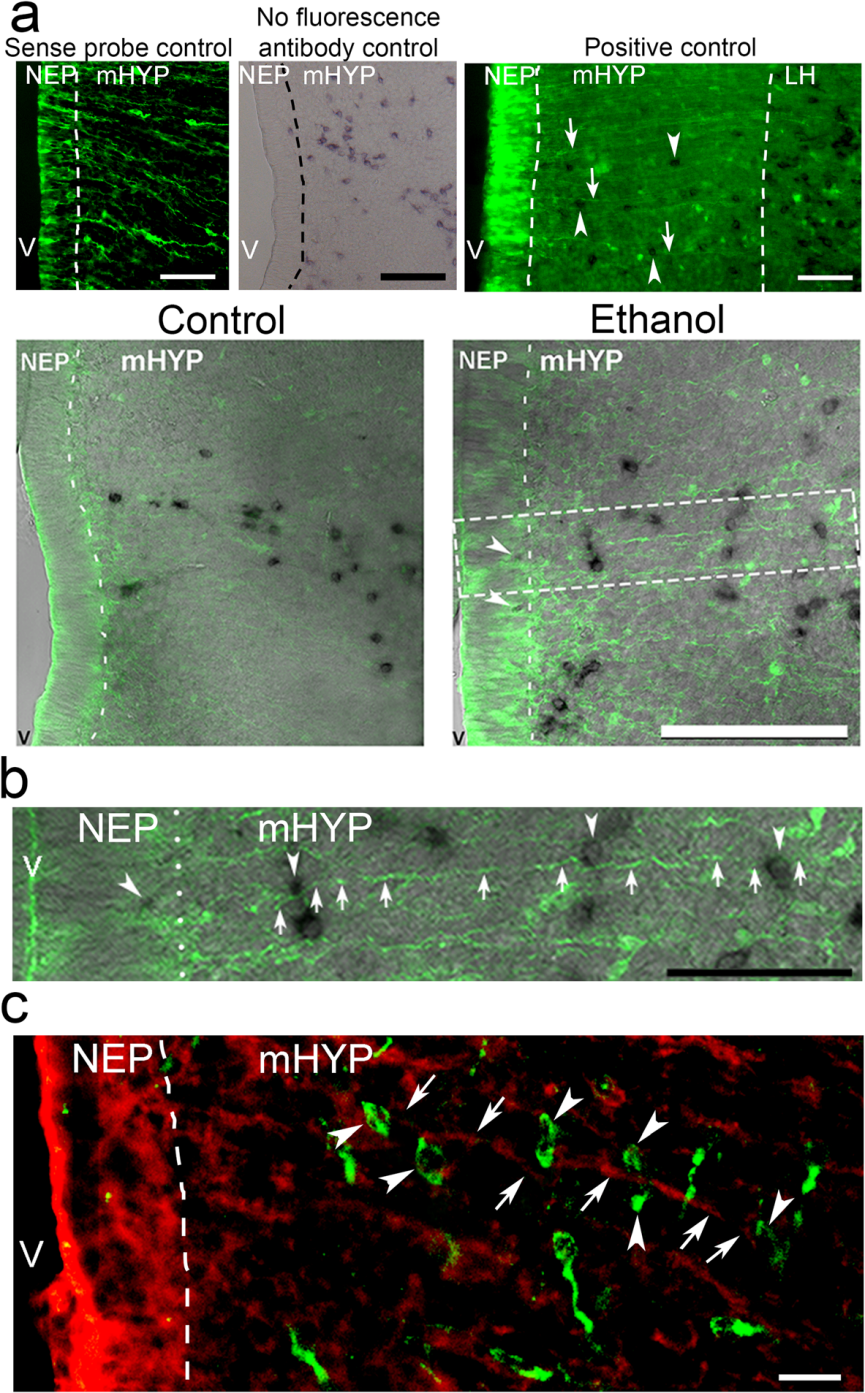
Increased MCH^+^ neurons closely related to radial glia cells and processes in NEP and mHYP. Photomicrographs illustrate how maternal ethanol administration (2 g/kg/day, E10–E15) compared to isocaloric Control group (*n* = 7/group) increases the number of MCH^+^ neurons in close relation to the radial glia cells and along their processes (measured by IF for BLBP^+^) in female E19 embryos, with the quantification and data described in detail in the “Methods” and “Results” section. **a** Top panel, negative controls (sense probe and no fluorescence antibody) and positive control (double-labeling of MCH DIG-ISH with BLBP IF) (× 10 regular fluorescence images). The sense probe control revealed no MCH-expressing neurons, and the no fluorescence antibody control revealed no BLBP immunofluorescent cells and processes. The positive control shows the MCH-expressing neurons (black) to be very dense in the LH, with some evident in the mHYP (indicated by white arrowheads) and a few in the NEP, and the BLBP-labeled cells (green) to be most concentrated in NEP while their processes (green) are densest in the mHYP (indicated by white arrows) as they project laterally toward the LH. Bottom panel, photomicrographs combining DIG-labeled in situ hybridization for MCH^+^ neurons and immunofluorescence histochemistry for BLBP^+^ radial glia (× 20 confocal images). In ethanol-exposed embryo compared to control, these images illustrate several MCH^+^ neurons (two indicated by white arrowheads) in NEP, not evident in Control, that lie lateral to the BLBP^+^ radial glia cells and the increased number and percentage of MCH^+^ neurons in mHYP that are positioned close to the radial glia processes as they project laterally toward LH (see text for data). **b** This close relationship between the MCH^+^ neurons and radial glia is more clearly illustrated in the enlarged image (from the area between dotted white lines in of ethanol-exposed embryo), showing one MCH^+^ neuron in the NEP and three MCH^+^ neurons (white arrowheads) along of a single BLBP^+^ process (white arrow) projecting laterally through the mHYP. **c** Further analyses using double-labeled immunofluorescence histochemistry confirmed this close relationship between embryonic MCH^+^ neurons (green) and radial glia (red) in the mHYP, with × 40 image showing six MCH^+^ neurons (white arrowheads) along a single BLBP^+^ process (white arrows) projecting toward the LH. BLBP, brain lipid-binding protein; mHYP, medial hypothalamus; MCH, melanin-concentrating hormone; NEP, neuroepithelium; V, third ventricle. Scale bars, 100 μm

We next determined whether the appearance and patterning of MCH^+^ neurons are linked to the ethanol-induced stimulation of radial glia cells and processes. We employed a combination of DIG-ISH to label MCH^+^ neurons and IF to label BLBP^+^ radial glia in female embryos of dams that received daily intraoral administration (from E10 to E15) of either 2 g/kg/day of ethanol (Ethanol) or an isocaloric maltose-dextrin control solution (Control), and quantified the MCH^+^ neurons as they relate to the radial glia (described in the “Methods” section). Replicating the results described above, we found Ethanol compared to Control to increase the density of MCH^+^ neurons in the NEP (*p* < 0.001) and the mHYP (*p* = 0.034) and also to increase the density of BLBP^+^ cells in the NEP (*p* = 0.026) and BLBP^+^ processes in the mHYP (*p* = 0.048) (Table 8). With the combination of DIG-ISH and BLBP IF, we discovered in female embryos from a separate set of dams that the individual MCH^+^ neurons are closely related to the radial glia in both the NEP and mHYP, as illustrated most clearly in the photomicrographs of ethanol-exposed embryos (Fig. 9). In the NEP where the MCH^+^ neurons are relatively sparse, Ethanol compared to Control group significantly increased their number along the edge of this midline region, from 0.0 to 4.14 ± 0.34 cells (*t*(12) = − 12.182, *p* < 0.001), in the area just lateral to the radial glia cells. Moreover, in the mHYP where the MCH^+^ neurons are more abundant, ethanol also significantly increased their number, from 112.0 ± 3.53 to 134.0 ± 5.31 cells (*t*(12) = − 3.359, *p* = 0.006), in the area of the radial glia processes. This increase occurred in both the number of MCH^+^ neurons, from 9.29 ± 0.61 to 21.00 ± 0.98 (*t*(12) = 1 10.20, *p* < 0.001), and the percentage of total MCH^+^ neurons, from 8.31 ± 0.53 to 16.02 ± 1.00% (*t*(12) = − 6.638, *p* < 0.001), that are positioned close to and along the BLBP^+^ processes as they project laterally through the mHYP toward the LH (Fig. 9a). These effects of ethanol are clearly evident in the enlarged image showing three MCH^+^ neurons (white arrowheads) along a single radial glia process (white arrows) in the mHYP (Fig. 9b). We confirmed this anatomical relationship between MCH^+^ neurons and a distinct BLBP^+^ process using double-labeled IF for BLBP and MCH and found in the mHYP and LH of an ethanol-exposed embryo a rich concentration of the radial glia processes (but few cells) and the MCH^+^ neurons (Fig. 9c). Many of these MCH^+^ neurons (green) were located close to the radial glia processes (red), as illustrated by the six neurons (white arrowheads) along a single BLBP^+^ process (white arrows). These results suggest that the stimulatory effect of ethanol on the density of MCH^+^ neurons originates within the embryonic NEP in close relation to the radial glia cells, and it involves the radial glia processes, along which the MCH neurons are positioned as they project laterally through the mHYP and continue into the LH, their final destination.

**Table 8:** Effects of ethanol on MCH and BLBP cells and processes in NEP and mHYP of female embryos

Labeled cells/processes	Location	Control (cells, objects/μm^2^)	Ethanol (cells, objects/μm^2^)
MCH^+^	NEP	3.77E− 6 ± 2.47E− 6	2.58E− 5 ± 2.18E− 6*
	mHYP	4.21E− 5 ± 1.89E− 6	4.92E− 5 ± 2.31E− 6*
BLBP^+^	NEP	1.09E− 4 ± 7.76E− 6	1.31E− 4 ± 6.44E− 6*
	mHYP	1.37E− 4 ± 6.99E− 6	1.65E− 4 ± 8.69E− 6*

Maternal administration of ethanol (2 g/kg/day, E10–E15) was compared to isocaloric maltose-dextrin control solution (Control) group in the NEP and mHYP areas of female embryos, measured using DIG-labeled in situ hybridization and immunofluorescence histochemistry. Data are mean ± SEM. *n* = 6/group, *p < 0.05 versus Control. One-way ANOVA was used to compare group means followed by SDS post hoc test.

*BLBP* brain lipid-binding protein, *MCH* melanin-concentrating hormone, *mHYP* medial hypothalamus, *NEP* hypothalamic neuroepithelium

## Discussion

Studies to date of radial glia have shown that prenatal exposure to ethanol, generally administered at chronic high doses, causes morphological and functional defects in these progenitor cells, decreasing their density and disrupting their function in promoting the differentiation and migration of neurons [30, 50]. Our results here, with ethanol administered at a moderate dose during a brief period of rapid neurogenesis, demonstrate a strong stimulatory effect on the expression and density of BLBP^+^ radial glia cells concentrated in the hypothalamic NEP along the third ventricle, suggesting that the level of mRNA per cell may be increased, and also on the expression and density of their processes projecting laterally through the mHYP and into the LH. These effects, indicating a site and mechanism through which increased neurogenesis and neuronal migration may be induced by ethanol, are consistent with in vitro studies showing relatively low concentrations to stimulate the appearance of radial glia-like precursors and the migration of differentiating neurons [51, 52].

Neuroimmune factors including chemokines in the brain are shown to be rich in the embryonic NEP and suggested to play a role in neural development [53]. Whereas most studies have focused on the inflammatory CXCL12/CXCR4 system and its function in stimulating neural stem cell differentiation and neuronal migration [54, 55], the CCL2/CCR2 system shown to be expressed in the embryonic hypothalamus [56] and LH [12] also appears to be involved. There is evidence that CCL2 in vitro stimulates the proliferation, differentiation, and migration of cultured neural progenitor cells [57, 58] and the migration of hypothalamic embryonic neurons in cell culture [59] and that knockout of the CCL2 gene disrupts neural progenitor cell migration [60]. We demonstrate here that CCL2 cells in E19 embryos are densely concentrated in the hypothalamic NEP along the third ventricular border with short processes extending laterally into the mHYP, and that these cells and processes are stimulated by moderate maternal administration of ethanol and similarly by maternal administration of CCL2 itself while blocked by a CCR2 antagonist. These new findings focus attention on the hypothalamic NEP as a site where neurons in the LH expressing CCL2 and CCR2 are born [11, 12]. Together with in vitro studies [61, 62] and our reports showing maternal CCL2 administration to increase CCL2 cell density in the NEP as shown here and in the LH [11, 12] and central injection of CCL2 to stimulate CCL2 in radial glia cells and processes in embryonic NEP [63], they reveal an auto-regulatory mechanism that involves an increase in the synthesis of CCL2 [63].

Our results also suggest that the fetal CCL2/CCR2 system promotes neuronal development as it functions within the radial glia neuroprogenitor cells in the hypothalamic NEP and their long processes projecting laterally through the mHYP and into the LH. They demonstrate that CCL2 colocalizes extensively with these BLBP^+^ radial glia cells and processes, consistent with evidence in the spinal cord [32], and that maternal ethanol at moderate levels markedly stimulates CCL2 in the radial glia and also in local neurons but not microglia, similar to that described in the LH in our prior studies [11, 12]. These new findings confirm the idea that CCL2 plays many roles during development as it is found in different cell types, including radial glia and neurons. Furthermore, the evidence presented in this report shows that these effects of maternal ethanol are similar to the effects of maternal administration of CCL2 and are reversed by a CCR2 antagonist supporting the idea that the CCL2/CCR2 system in the hypothalamic NEP when stimulated by ethanol in the embryo functions synergistically with and within the radial glia neuroprogenitor cells and processes to promote the differentiation and migration of neurons, including those shown to express CCL2 or CCR2 in the LH [12]. With BLBP in addition to being a radial glia marker also shown to be required for radial elongation [64], a possible involvement of BLBP itself in these stimulatory effects of ethanol and CCL2 on the radial glia processes cannot be excluded.

Our analyses of the MCH neurons show them to be closely related to the radial glia, both their cells and processes, in the E19 embryo. In this report, maternal ethanol administration from E10 to E15, which overlaps the period of MCH neurogenesis [65], increases both mRNA expression and density of MCH neurons in the NEP immediately lateral to the radial glia progenitor cells from which most hypothalamic neurons are suggested to originate [24]. These findings again focus attention on the embryonic NEP, specifically on its CCL2-expressing radial glia cells, as a site highly sensitive to ethanol’s inflammatory actions and a source of the increased MCH neurons detected locally. With radial glial processes known to provide scaffolding in the embryo to facilitate the migration of neurons to outer brain regions [29, 66], our additional findings here that ethanol increases the number of MCH neurons positioned along and in close contact with the long CCL2-colocalizing radial glia processes projecting laterally through the mHYP support their role in guiding these neuroepithelial peptide neurons toward their final destination, which is mainly the LH [67]. With radial glia progenitor cells highly active in the embryo but lost shortly after birth [68] and ethanol’s effects on glial precursor cells found to cause long-term changes in neuronal development and function [6], ethanol-induced increase in CCL2-rich radial glia progenitor cells and processes described in this report is likely to be an important component of the early developmental phenomena ultimately leading to the increased density in postnatal and adolescent offspring of MCH neurons in LH that co-express CCL2 and CCR2 [11, 12, 15].

The sexually dimorphic nature of ethanol’s stimulatory effects in the NEP, with greater changes consistently observed in female embryos, is of particular interest since it was previously observed in LH in our prior studies where maternal ethanol similarly stimulated CCL2/CCR2 system and MCH neurons in the embryo and adolescent offspring [11, 12, 15]. The most notable finding in this report is that CCL2/CCR2 system in the NEP while strongly stimulated by ethanol in female embryos is totally unresponsive in males, a stark contrast that is consistent with measurements of gene expression reported in the fetal cortex and hippocampus [69]. While there is limited evidence suggesting sexual dimorphism in neuroimmune systems that affect neuronal differentiation [69–71], the dramatic sex differences revealed here in the embryo, possibly reflecting effects of sex hormones and enzymes known to stimulate neurogenesis [72, 73], focus attention on the CCL2/CCR2 system in neuroprogenitor cells of the hypothalamic NEP as a key mechanism underlying ethanol’s sexually dimorphic, stimulatory effects on neuronal development. This includes the birth and migration of neurons expressing CCL2, CCR2, and MCH that become concentrated in the LH and remain increased even in adolescent offspring [11, 15].

## Conclusions

**Fig. 10:**
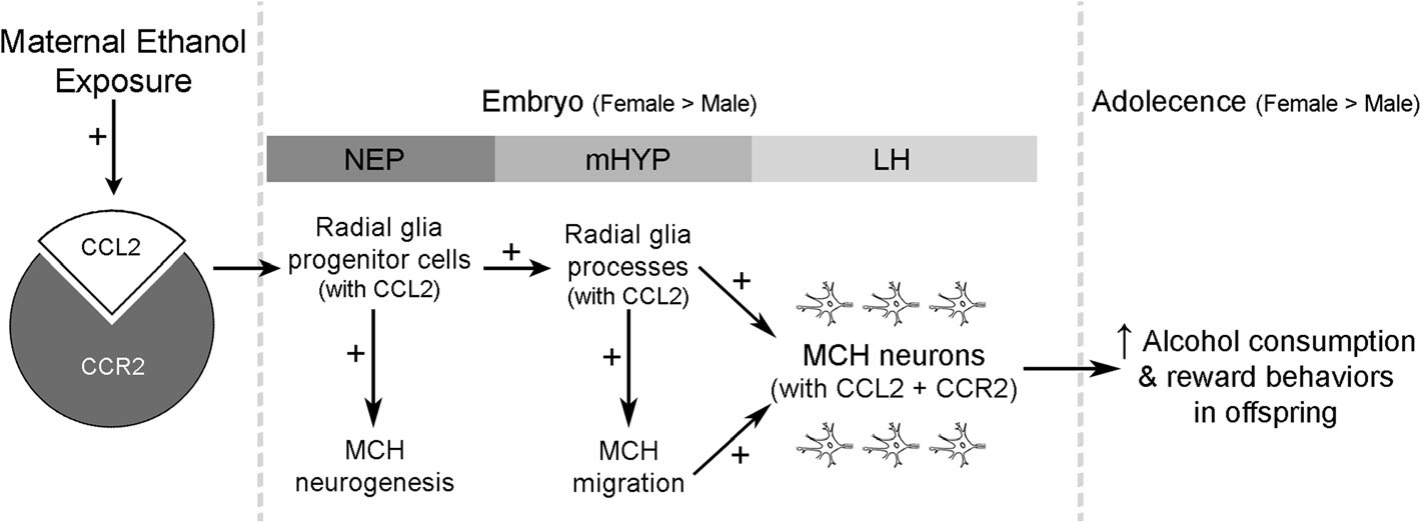
Schematic representation of proposed neuroimmune mechanism underlying the stimulatory effect of maternal ethanol exposure at a moderate dose on early development of MCH neurons and ultimately on the offspring’s behavior. Maternal ethanol exposure (2 g/kg/day, E10–E15) increases CCL2 signaling at its receptor CCR2, which in turn stimulates radial glia progenitor cells with CCL2 in the NEP that function to promote MCH neurogenesis in the NEP and also radial glia processes with CCL2 in the mHYP that facilitate the migration of MCH neurons toward the LH where they themselves colocalize CCL2 and CCR2. These molecular changes in the embryo, consistently stronger females than males, may contribute to the increased alcohol consumption and reward behaviors observed later in adolescent offspring, often more strongly in females. LH, lateral hypothalamus; MCH, melanin-concentrating hormone; mHYP, medial hypothalamus; NEP, neuroepithelium

In summary, the results of this study suggest a novel mechanism in the embryo through which maternal ethanol administration at low-to-moderate doses causes lasting alterations in neuronal systems of the offspring. This mechanism, schematically illustrated in Fig. 10, involves the stimulation of CCL2/CCR2 signaling and radial glia neuroprogenitor cells in the NEP that function together to promote the development of MCH neurons and also the stimulation of CCL2-expressing radial glia processes in the mHYP that facilitate the migration of MCH neurons toward the LH where they colocalize CCL2 and CCR2. Further fate-mapping studies of this embryonic neural mechanism in the hypothalamus, as described for microglia [74], should help to determine the precise origin of the MCH neurons from CCL2-expressing radial glia and characterize in greater depth the mechanisms through which early ethanol exposure directly impacts neuronal development. These findings in the embryo have important clinical significance. Studies in humans as well as animals show maternal ethanol consumption and neuroinflammation during pregnancy to increase alcohol intake in the offspring [2, 4, 75] and suggest a role for MCH in reward, alcohol overconsumption, and increased risk for alcohol abuse [18, 76]. With MCH also known to have a significant role in the sleep/wake cycle [77], this neuropeptide when stimulated by maternal ethanol exposure may contribute to the disturbances in sleep commonly associated with alcohol use disorder [78]. Further, evidence that the ethanol-induced effects on MCH neurons as well as behavior occur more strongly in females than males [11, 12, 15] and MCH in females but not males is expressed in the laterodorsal tegmentum [79] that is involved in reward-related behavior [80], may have implications for understanding the increased risk factors for alcohol abuse described in women [81, 82]. Together, this evidence further supports MCH as a potential therapeutic target for preventing the emergence and relapse of alcohol use disorder [13, 17].

## Data Availability

The datasets used and/or analyzed during the current study are available from the corresponding author on reasonable request.

## Abbreviations

MCH: Melanin-concentrating hormone
LH: Lateral hypothalamus
CCL2: Chemokine C-C motif ligand 2
CCR2: CCL2 receptor
E10, E15, E19: Embryonic day 10, embryonic day 15, embryonic day 19
qRT-PCR: Quantitative real-time polymerase chain reaction
NEP: Neuroepithelium
mHYP: Medial hypothalamus
IF: Immunofluorescence histochemistry
BLBP: Brain lipid-binding protein
NeuN: Neuronal marker
Iba-1: Microglial marker
CXCL12: C-X-C motif chemokine 12
CXCR4: C-X-C chemokine receptor type 4
